# Lcp1 Is a Phosphotransferase Responsible for Ligating Arabinogalactan to Peptidoglycan in *Mycobacterium tuberculosis*

**DOI:** 10.1128/mBio.00972-16

**Published:** 2016-08-02

**Authors:** James Harrison, Georgina Lloyd, Maju Joe, Todd L. Lowary, Edward Reynolds, Hannah Walters-Morgan, Apoorva Bhatt, Andrew Lovering, Gurdyal S. Besra, Luke J. Alderwick

**Affiliations:** a Institute of Microbiology and Infection, School of Biosciences, University of Birmingham, Birmingham, United Kingdom; b Alberta Glycomics Centre and Department of Chemistry, University of Alberta, Gunning-Lemieux Chemistry Centre, Edmonton, Canada

## Abstract

*Mycobacterium tuberculosis*, the etiological agent of tuberculosis (TB), has a unique cell envelope which accounts for its unusual low permeability and contributes to resistance against common antibiotics. The main structural elements of the cell wall consist of a cross-linked network of peptidoglycan (PG) in which some of the muramic acid residues are covalently attached to a complex polysaccharide, arabinogalactan (AG), via a unique α-l-rhamnopyranose–(1→3)-α-d-GlcNAc-(1→P) linker unit. While the molecular genetics associated with PG and AG biosynthetic pathways have been largely delineated, the mechanism by which these two major pathways converge has remained elusive. In Gram-positive organisms, the LytR-CpsA-Psr (LCP) family of proteins are responsible for ligating cell wall teichoic acids to peptidoglycan, through a linker unit that bears a striking resemblance to that found in mycobacterial arabinogalactan. In this study, we have identified *Rv3267* as a mycobacterial LCP homolog gene that encodes a phosphotransferase which we have named Lcp1. We demonstrate that *lcp1* is an essential gene required for cell viability and show that recombinant Lcp1 is capable of ligating AG to PG in a cell-free radiolabeling assay.

## IMPORTANCE

Tuberculosis is an infectious disease caused by the bacterial organism *Mycobacterium tuberculosis*. Survival of *M. tuberculosis* rests critically on the integrity of its unique cell wall; therefore, a better understanding of how the genes and enzymes involved in cell wall assembly work is fundamental for us to develop new drugs to treat this disease. In this study, we have identified Lcp1 as an essential phosphotransferase that ligates together arabinogalactan and peptidoglycan, two crucial cell wall macromolecules found within the mycobacterial cell wall. The discovery of Lcp1 sheds new light on the final stages of mycobacterial cell wall assembly and represents a key biosynthetic step that could be exploited for new anti-TB drug discovery.

## INTRODUCTION

Tuberculosis (TB) remains the single most important bacterial cause of global mortality and morbidity, causing around 9.6 million new cases and 1.5 million deaths each year (1). This prevalence is largely due to the increased susceptibility of HIV-infected individuals, and the rise of multidrug-resistant (MDR), extensively drug-resistant (XDR), and, more recently, totally drug-resistant (TDR) TB strains (2). It is of paramount importance that we extend our understanding of the pathogenicity and physiology of the tubercle bacillus in the hope of priming novel therapeutic approaches against this ancient human adversary. Mycobacterial peptidoglycan (PG) forms the basal layer of the mycolyl-arabinogalactan–peptidoglycan (mAGP) complex and is composed of alternating *N*-acetylglucosamine (GlcNAc) and modified muramic acid (Mur) residues, linked in a β(1→4) configuration (3). Unlike archetypal PG commonly found in bacteria such as *Escherichia coli*, the muramic acid residues in *Mycobacterium tuberculosis* contains a mixture of N-acetyl and N-glycolyl derivatives, whereby the N-acetyl function has been oxidized to an N-glycolyl function to form MurNGly (4–6). In addition, approximately 10 to 12% of the muramic acid residues of PG are covalently tethered to arabinogalactan (AG) via a phosphodiester bond (7). The 6′-OH groups of these muramic acid residues serve as attachment sites forming a phosphodiester bond via a unique α-l-rhamnopyranose–(1→3)-α-d-GlcNAc-(1→P) linker unit (LU) (8). Collectively, PG and AG form a huge macropolymer positioned between the cytoplasmic membrane and the outer mycolic acid layer of the TB bacillus. Our understanding of both the structure and biosynthesis of mycobacterial AG has developed steadily over the last 2 decades, with much emphasis being placed on investigating the molecular genetics of how this complex structure is assembled (extensively reviewed in reference 9). While the cytoplasmic and extracytoplasmic intermediate biosynthetic steps of cell wall assembly have been almost fully delineated, our understanding of the latter stages of AG biosynthesis, specifically the attachment of AG to PG, remains fragmented at best. Previous studies have demonstrated that attachment of AG to the mycobacterial cell wall occurs only when the PG has been fully matured via the cross-linking action of endogenous transpeptidases (10). However, the enzyme responsible for this action remains elusive. In Gram-positive bacteria, the structure of the linker unit (d-GlcNAc-d-ManNAc) that connects wall teichoic acids (WTA) to PG via a phosphodiester bond is remarkably similar to the linker unit found within the *M. tuberculosis* cell wall (d-GlcNAc-l-Rha*p*) (11). Other commonalities also exist between mycobacterial and Gram-positive cell wall glycan biosynthetic pathways. For instance, in organisms such as *Staphylococcus aureus* polyribitol-phosphate (Rbo-P) is assembled upon a polyprenyl pyrophosphoryl lipid carrier which, after biosynthesis is completed, is ultimately attached to the C_6_-hydroxyl of MurNAc within the PG glycan strands (8, 11). Mycobacterial AG is also assembled upon a polyprenyl pyrophosphoryl lipid carrier before its final deposition onto specific muramic acid residues of PG (12). We postulated that WTA-containing Gram-positive organisms and members of the *Actinomycetales* might utilize similar ligases from the same protein superfamily. Kawai et al. conducted a structural and genetic study and proposed that a family of genes encoding the LytR-CpsA-Psr (LCP) proteins catalyze the ligation of the murine linkage unit of WTA to the MurNAc units of PG in *Bacillus subtilis* (13). More recent studies have revealed further corroborating evidence that the LCP family of proteins are responsible for attaching capsular polysaccharides to the murine sacculus of *S. aureus* (14) and *Streptococcus pneumoniae* (15). Here, we demonstrate that the open reading frame contained in *Rv3267*, one of three LCP ortholog genes in *M. tuberculosis*, encodes a peptidoglycan–arabinogalactan ligase, which we have termed Lcp1.

## RESULTS

### Genome comparison of the Lcp1 locus.

**FIG 1:**
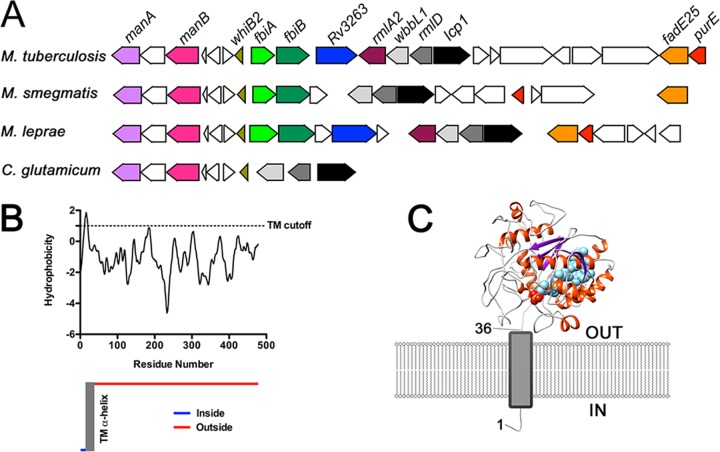
Comparison of the *lcp1* locus and *in silico* analysis of Lcp1 by TMHMM and I-TASSER. (A) The locus in bacteria analyzed consists of *lcp1*, which in *M. tuberculosis* has the locus tag *Rv3267* and in *M. smegmatis* has the locus tag *MSMEG1824*. For *M. leprae* and *C. glutamicum*, locus tags are *ML0756* and *NCgl0708*, respectively. (B) The TMHMM server was used to predict the membrane topology of ^Mtb^Lcp1 (48). (C) I-TASSER homology model of ^Mtb^Lcp1 positioned in relation to the cytoplasmic membrane and predicted N-terminal TM α-helix (19).

Using *S. aureus*, *S. pneumoniae*, and *B. subtilis* LCP proteins as search sequences, we performed a bioinformatic analysis of mycobacterial and corynebacterial genomes to identify putative genes that might encode the mycobacterial LCP homologs (see Fig. S1 in the supplemental material). We identified three putative LCP homologs in *M. tuberculosis*, with Rv3267 sharing 20% amino acid sequence identity with Cps2A from *S. pneumoniae*, which is in accordance with a previous phylogenetic study of bacterial LCP proteins (16). The remaining two putative LCP orthologs present in *M. tuberculosis*, encoded by *Rv3484* and *Rv0822c*, share 15% and 12% amino acid identity with Cps2A from *S. pneumoniae*. Rv3267 shares 36% and 26% amino acid sequence identity with Rv3484 and Rv0822c, respectively. Both Rv3484 and Rv0822c are predicted to contain a single N-terminal transmembrane (TM) α-helix, similar to that of Rv3267, and are predicted to contain domains belonging to both LCP and LytR_C superfamilies. Interestingly, the genome of *Mycobacterium leprae*, which shows massive gene decay and is thus considered to represent a “minimal” mycobacterial genome, has retained only two LCP orthologs in the form of ML0750 and ML2247, sharing 79.1% and 32.7% amino acid sequence identity with Rv3267, respectively. Inspection of the *Mycobacterium smegmatis* genome reveals four putative orthologs, MSMEG_1824, MSMEG_6421, MSMEG_0107, and MSMEG_5775, each sharing amino acid sequence identities of 72%, 35%, 31%, and 24% with Rv3267, respectively. Furthermore, an alignment of the genes surrounding *Rv3267* reveals genetic synteny with orthologous regions from *M. smegmatis*, *M. leprae*, and *Corynebacterium glutamicum* (Fig. 1A)*.* This close arrangement of genes is highly indicative of conserved functionality. Interestingly, *Rv3267* is positioned immediately upstream of *rmlD* and *wbbL1*, both of which encode enzymes responsible for the formation of the linker unit during early-stage AG biosynthesis (Fig. 1A) (17, 18). Based on these analyses, we hypothesized that Rv3267 was the primary LCP homolog responsible for ligating PG to AG in mycobacteria, which we have named Lcp1.

### *In silico* analysis of ^Mtb^Lcp1 and identification of copurifying polyisoprenoid phosphate.

**FIG 2:**
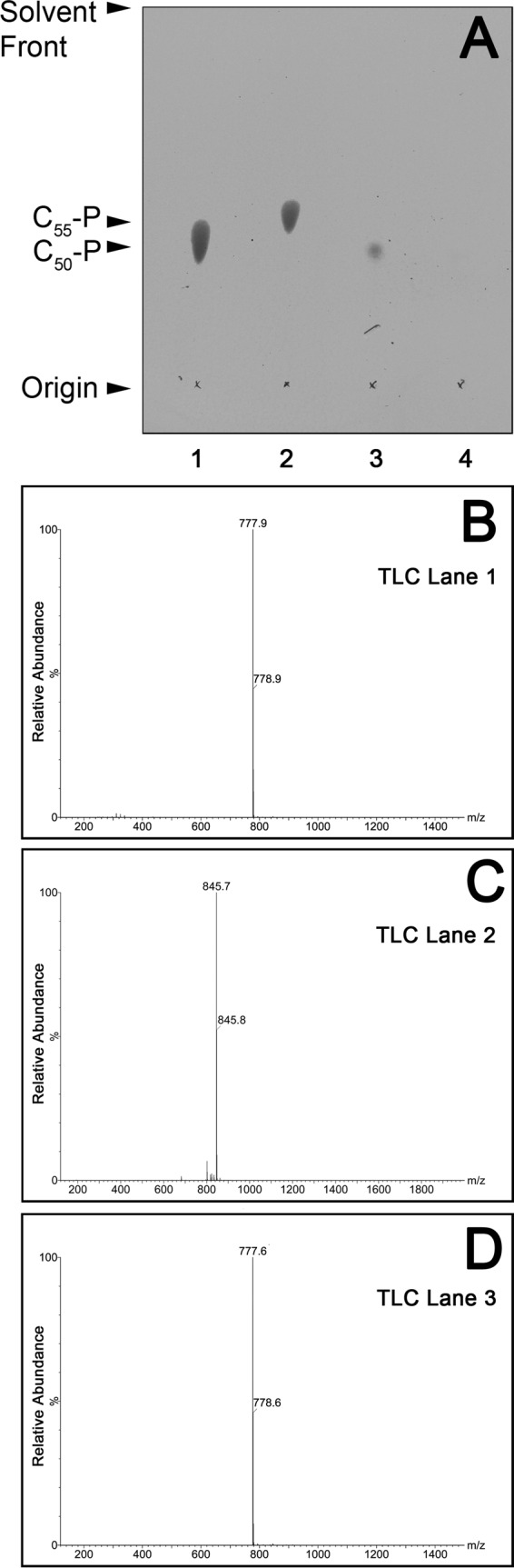
TLC and ES-MS analysis and identification of decaprenyl-1-monophosphate bound to recombinant ^Mtb^Lcp1. (A) TLC analysis of organic extractable material of recombinant ^Mtb^Lcp1. Standards of decaprenyl-1-monophosphate and undecaprenyl-1-monophosphate are in lanes 1 and 2, respectively. Lane 3 contains material extracted from ^Mtb^Lcp1, while lane 4 contains a negative control of material extracted from EmbC^CT^ (21). Lipids were analyzed by TLC using silica gel plates (5735 silica gel 60F254; Merck) and developed in CHCl_3_-CH_3_OH-NH_4_OH-H_2_O (65:25:0.5:3.6, vol/vol/vol/vol), and plates were sprayed with 5% ethanolic molybdophosphoric acid and charred to visualize the lipids. (B) ES-MS analysis of decaprenyl-1-monophosphate standard (TLC, lane 1). (C) ES-MS analysis of undecaprenyl-1-monophosphate standard (TLC, lane 2). (D) ES-MS organic extracted material from ^Mtb^Lcp1 (TLC, lane 3).

Analysis of the *M. tuberculosis* Lcp1 (^Mtb^Lcp1) amino acid sequence suggests that a single transmembrane-spanning α-helix occurs at the N terminus of the protein (Fig. 1B). Similarly, a single N-terminal transmembrane α-helix has also been observed in other LCP proteins, such as Cps2A from *S. pneumoniae* (13, 16). An alignment of the amino acid sequences of ^Mtb^Lcp1 and Cps2A from *S. pneumoniae* indicates that these two proteins share 20% sequence identity (see Fig. S1 in the supplemental material). The crystal structure of ΔTM-Cps2A (residues 98 to 481) has been solved to a resolution of 1.69 Å with one molecule of decaprenol-1-monophosphate bound in a central hydrophobic cavity (2XXP) (13). Although we were able to express large amounts of pure, soluble His6-tagged ^Mtb^Lcp1 (see below), our attempts to obtain an experimental crystal structure of ^Mtb^Lcp1 have so far proven unsuccessful. Therefore, we generated an ^Mtb^Lcp1 homology model using the I-TASSER server (utilizing the *S. pneumoniae* Cps2A structure as the template), resulting in a C-score of −2.64 (19, 20). Unlike the *S. pneumoniae* ΔTM-Cps2A structure, which also harbors an N-terminal accessory domain, the ^Mtb^Lcp1 amino acid sequence and homology model suggests that only a single “LCP-like” domain is present (Fig. 1C) (13). Our ^Mtb^Lcp1 homology model indicates that a 5-stranded β-sheet forms the core of the protein, with α-helices surrounding a central β-sheet. Our homology model also retains the polyisoprenoid-binding cavity of Cps2A (Fig. 1C) (13). Overexpression of His6-tagged ^Mtb^Lcp1 in *E. coli* BL21(DE3) cells and subsequent purification by immobilized metal affinity chromatography (IMAC) resulted in a preparation of His6-tagged ^Mtb^Lcp1 that was stable in solution at a concentration of 50 mg/ml. Since Cps2A was shown to copurify with a polyisoprenoid phosphate, we also investigated whether His6-tagged ^Mtb^Lcp1 copurified with a similar lipid (13). We performed an organic solvent extraction on His6-tagged ^Mtb^Lcp1 followed by thin-layer chromatography (TLC) analysis, which revealed a spot that migrated with an *R_f_* identical to that of decaprenyl-1-monophosphate (Fig. 2A). Mass spectrometric analysis of this copurifying lipid revealed an *m/z* of 777, which correlates with the expected mass of decaprenyl-1-monophosphate (Fig. 2D). This evidence was further corroborated by comparison to known standards of decaprenyl-1-monophsophate and undecaprenyl-1-monophsophate, which reveal *m/z* values of 777 and 845, respectively (Fig. 2B and C). We also performed an identical solvent extraction on a highly purified preparation of EmbC^CT^ (the C-terminal globular domain of the arabinofuranosyltransferase EmbC from *M. tuberculosis* [21]) (Fig. 2A). This negative control confirms that the product extracted from ^Mtb^Lcp1 is not a contaminating impurity from the extraction process.

### ^Ms^Lcp1 is essential for viability of *M. smegmatis*.

**FIG 3:**
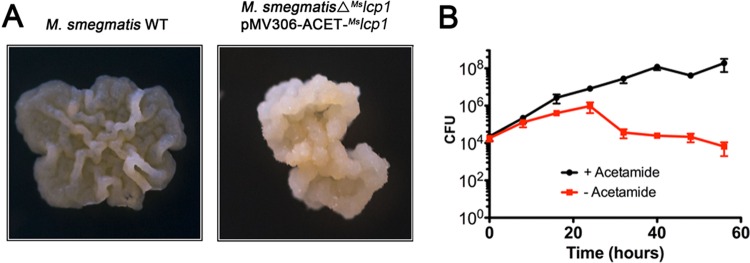
Essentiality of *^Ms^*lcp1 in *M. smegmatis* mc^2^1551. (A) Growth and colony morphology of *M. smegmatis* and Δ*^Ms^*lcp1 conditional mutant on tryptic soy agar with the inducer acetamide. WT, wild type. (B) CFU counts of *M. smegmatis* Δ*^Ms^*lcp1 conditional mutant cultured in tryptic soy broth in the presence and absence of the inducer acetamide (data plotted represent the mean ± standard deviation from three independent biological replicate experiments).

Because of its putative role in covalently attaching AG to PG in mycobacteria, we hypothesized that *^Mtb^*lcp1 would likely be an essential gene. In order to test this hypothesis, we used *M. smegmatis* as a model system to investigate the essentiality of Lcp1 in mycobacteria using the conditional expression specialized transduction essentiality test (CESTET) (22–25). We constructed a knockout phage, phΔ^Ms^lcp1, designed to replace *MSMEG1824* (*M. smegmatis lcp1* [*^Ms^*lcp1]) with a hygromycin resistance cassette (22), but as predicted, we were unable to directly generate a null mutant due to the failure to yield any transductants. However, we were able to generate an *^Ms^*lcp1 null mutant (*M. smegmatis* Δ^Ms^lcp1 pMV306-ACET-^Ms^lcp1) by transducing a merodiploid strain containing a second, inducible copy of *^Ms^*lcp1. Expression of this recombinant copy of *^Ms^*lcp1 was induced by the addition of acetamide to the growth medium, and only when this inducer was present on agar plates were we able to generate conditional knockout mutants. In this regard, the conditional knockout mutant (*M. smegmatis* Δ^Ms^lcp1 pMV306-ACET-^Ms^lcp1) exhibited an altered colony phenotype compared to the wild type, which is suggestive of a defect or lesion in the cell wall, which might not be fully recovered by complementation via the acetamide-inducible pMV306-ACET-^Ms^lcp1 plasmid (Fig. 3A). To further investigate the essentiality of *^Ms^*lcp1, we cultured *M. smegmatis* Δ^Ms^lcp1 pMV306-ACET-^Ms^lcp1 in liquid medium and withdrew the inducer acetamide from the growth medium, with subsequent measurement of CFU. We observed a rapid loss of cell viability immediately after acetamide was removed from growth medium, indicating that expression of the pMV306-ACET-driven copy of *^Ms^*lcp1 was required for cell growth (Fig. 3B). This confirms that *^Ms^*lcp1 is indeed essential in *M. smegmatis*. The rapid loss in cell viability of the Δ*^Ms^*lcp1 mutant made any attempt to characterize the resultant phenotype (specifically cell wall lesions) technically challenging. Therefore, we turned our attention toward the biochemical characterization of recombinant ^Mtb^Lcp1.

### Carbohydrate binding of linker unit mimetics to ^Mtb^Lcp1.

**FIG 4:**
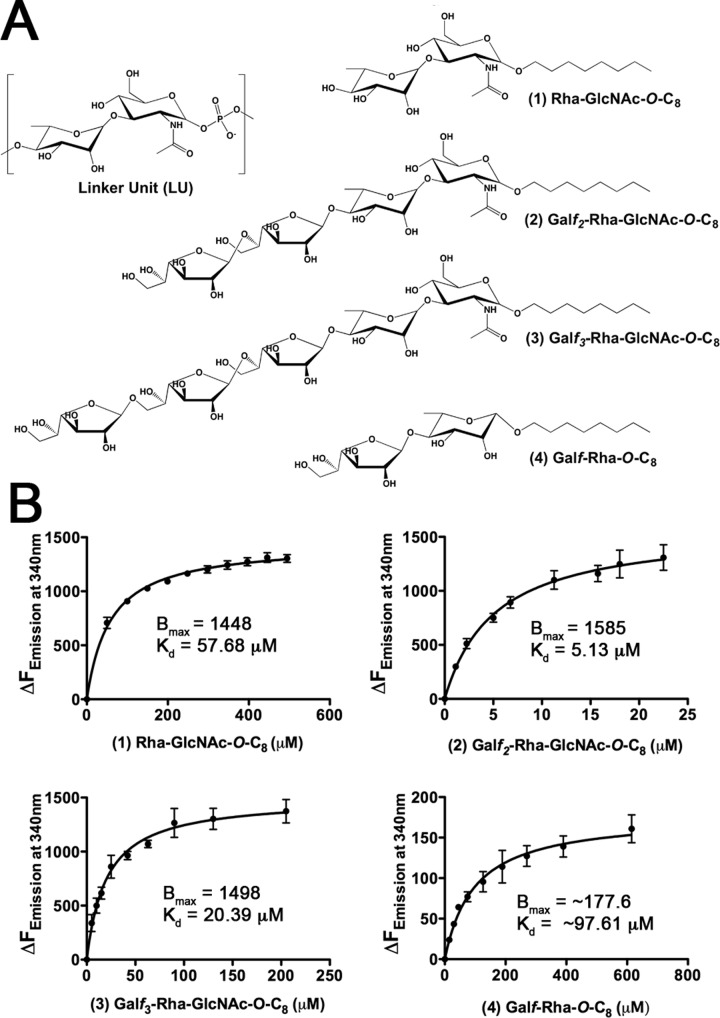
Binding of linker unit analogues to ^Mtb^Lcp1. (A) Chemical structure of the mycobacterial linker unit and novel chemical scaffolds used as ligands to probe the interaction of the linker unit with ^Mtb^Lcp1. (B) Saturation binding experiments using intrinsic tryptophan fluorescence of ^Mtb^Lcp1 to study binding of ligands 1 to 4. Data plotted represent the mean ± standard deviation from three independent biological replicate experiments.

Our laboratory has previously synthesized neoglycolipid acceptors modeled on motifs found within mycobacterial AG, which have been successfully employed to probe both enzymatic function and ligand binding relationships of cell wall processing enzymes (21, 26, 27). In this regard, we synthesized a panel of analogues of the mycobacterial linker unit (compounds 1 to 4) (Fig. 4A; see also Text S1 in the supplemental material) in order to probe the interaction of ^Mtb^Lcp1 with decaprenyl-pyrophosphoryl-GlcNAc-Rha-Galactose, its natural physiological substrate. Each of the ligands used (compounds 1 to 4) was synthesized to include an octyl (C_8_) group attached to the anomeric carbon. Historically, these alkyl groups are useful for performing solvent extractions from glycosyltransferase assay mixtures when used as neoglycolipid acceptors (26). In this study, their sole use was to mimic the hydrophobicity of the lipid moiety of the natural substrate, which would be accommodated by decaprenyl-pyrophosphate. Due to the chemical complexity and labile nature of the natural substrate (12, 28), using synthetic analogues offered us an alternative approach to investigate the biochemistry of this key enzymatic step. By way of intrinsic tryptophan fluorescence (ITF), we probed the binding of four potential ligands (compounds 1 to 4) to ^Mtb^Lcp1, each of which is a variant of the mycobacterial linker unit (Fig. 4A). ITF is an extremely useful approach to study protein-ligand interactions (29) and has been successfully used by our laboratory to biochemically characterize several mycobacterial proteins (21, 27, 30, 31). Fitting the binding data to a single-site saturation model yielded an equilibrium dissociation constant, *K_D_*, of 57.68 µM for compound 1, the ligand which resembles the mycobacterial linker unit disaccharide l-Rha-α(1→3)-d-GlcNAc (Fig. 4B). ITF experiments repeated with a tetrasaccharide ligand that resembles an extension of the linker unit by two Gal*f* residues afforded an ~10-fold increase in affinity, with a *K_D_* of 5.13 µM. Further elongation with a third Gal*f* unit resulted in a calculated *K_D_* of 20.39 µM. The cytoplasmic steps of mycobacterial galactan formation proceed via a linear biosynthetic pathway; therefore, compounds 2 and 3 represent chemical mimetics of glycolipid-3 (GL-3) and glycolipid-4 (GL-4) intermediates of this pathway, respectively (12, 28), without the pyrophosphoryl-decaprenyl moiety linked to the anomeric position of the GlcNAc unit. In addition, we investigated the binding of a fourth ligand against ^Mtb^Lcp1. Compound 4, a disaccharide of d-Gal*f*-β(1→4)-l-Rha*p* (27), mimics the terminal component of the linker unit with a single Gal*f* residue and displays an apparent *K_D_* of 97.61 µM. However, compound 4 achieves a *B*_max_ of only 177, which is significantly lower than those of compounds 1 to 3 and reflects the changes in fluorescence observed upon addition of equivalent volumes of water. Therefore, compound 4 displays no significant binding affinity (above background) toward ^Mtb^Lcp1 in ITF assays (Fig. 4B).

### ^Mtb^Lcp1 ligates newly synthesized galactan to peptidoglycan in a cell-free assay.

**FIG 5:**
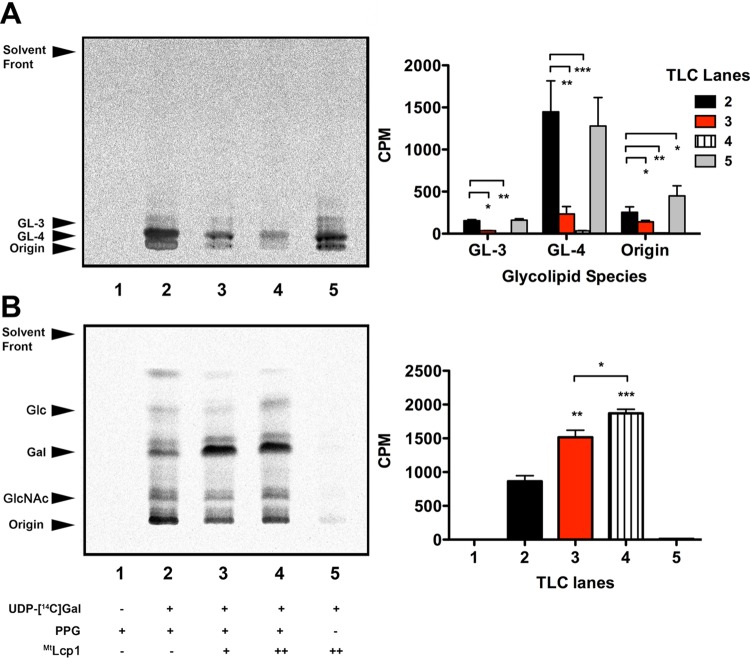
AG-PG ligase activity of ^Mtb^Lcp1 using a radiolabeled cell-free functional assay. (A) Organic solvent-extracted fractions from ^14^C-labeled assay mixtures were analyzed by TLC and developed in CHCl_3_-CH_2_OH-H_2_O-NH_4_OH (65:25:3.6:0.5, vol/vol/vol/vol) on aluminum-backed silica gel plates (5735 silica gel 60F254; Merck), and products were visualized by autoradiography, exposing the TLCs to X-ray film (Kodak X-Omat). TLC is representative of three independent biological replicate experiments, and corresponding densitometric data plotted represent the mean ± standard deviation for each band. Associated *P* values are as follows: *, *P* ≤ 0.05; **, *P* ≤ 0.01; ***, *P* ≤ 0.001. (B) Insoluble material from fraction 2 of the assay mixtures was hydrolyzed in 2 M TFA, reduced, and per-*O*-acetylated. The resulting ^14^C-labeled per-*O-*acetylated alditol acetate derivatives were analyzed by TLC using ethyl acetate-hexane (4:6, vol/vol) on aluminum-backed silica gel plates (5735 silica gel 60F254; Merck), and products were visualized by autoradiography, exposing the TLCs to X-ray film (Kodak X-Omat), and compared to known alditol-acetate standards of d-galactose (Gal), d-glucose (Glc), and d-*N*-acetylglucosamine (GlcNAc). TLC is representative of three independent biological replicate experiments, and corresponding densitometric data plotted represent the mean ± standard deviation for each band. Associated *P* values are as follows: *, *P* ≤ 0.05; **, *P* ≤ 0.01; ***, *P* ≤ 0.001.

We sought to investigate whether ^Mtb^Lcp1 is capable of ligating AG to PG (from decaprenyl-pyrophosphoryl-GlcNAc-Rha-galactose) by developing a cell-free biochemical functional assay. Since ^Mtb^Lcp1 contains a single N-terminal TM α-helix, and attachment of AG to PG is likely to occur at or around the cytoplasmic membrane, we postulated that membranes prepared from mycobacteria should contain endogenous PG-AG ligase activity and, as such, could be investigated using an appropriate [^14^C]carbohydrate radiolabeling assay. We have previously developed a radiolabel assay for monitoring the synthesis of decaprenyl-pyrophosphoryl-linked intermediates of mycobacterial cell wall biosynthesis (28). In this study, we modified this assay system so that we could directly investigate the *in vitro* covalent attachment of AG intermediates to PG. Briefly, membranes and the P60 fraction from *M. smegmatis* (both rich in endogenous cell wall biosynthetic activity) were combined with substrates in the form of purified peptidoglycan (PPG) and UDP-[^14^C]Gal*p*. The endogenous mutase activity within the P60 fraction of this assay mix (used to convert UDP-[^14^C]Gal*p* to UDP-[^14^C]Gal*f*) was sufficient to allow direct incorporation of [^14^C]Gal*f* into cell wall intermediates, as previously demonstrated (12, 28). PPG is a highly purified preparation of nascent *M. smegmatis* PG free from AG covalently attached to the 6′-OH position of MurNAc via a phosphodiester bond (32). The purpose of PPG is both to provide a substrate for attachment of ^14^C-labeled linker unit-galactan intermediates and to serve as a “vehicle” for extraction from the assay mixture for subsequent radiochemical quantification. Finally, the assay mixture was initiated by addition of highly purified recombinant ^Mtb^Lcp1. The analytical strategy for the products formed from this assay is illustrated as a flow chart in Fig. S4 in the supplemental material. Analysis of the fraction containing the CHCl_3_-CH_3_OH organic extraction by TLC reveals three major bands migrating toward the bottom of the TLC plate (Fig. 5A, lane 2). These bands correspond to ^14^C-labeled decaprenyl-P-P-GlcNAc-Rha-[^14^C]Gal*f* (GL-3) and decaprenyl-P-P-GlcNAc-Rha-[^14^C]Gal*f*_2_ (GL-4), with decaprenyl-P-P-GlcNAc-Rha-[^14^C]galactan remaining at the origin of the TLC plate (12, 28). Interestingly, the intensity of each of these bands diminished in a titration-dependent response as the amount of ^Mtb^Lcp1 increased in the assay mix (Fig. 5A, lanes 3 and 4), which was also quantified by densitometric analysis. When we repeated the assay at the highest ^Mtb^Lcp1 concentration in the absence of purified PG (PPG), we observed a product profile on the TLC with band intensities almost identical to that of the control reaction (Fig. 5A, lane 5). These data clearly demonstrate that upon addition of ^Mtb^Lcp1 to the assay mixture, the relative amount of [^14^C]Gal*f*-labeled material is reduced in the extracted organic fraction. We continued our analysis of the assay products by turning our attention to the insoluble fraction that contains PG and PG-linked [^14^C]Gal*f*-labeled material. Complete acid hydrolysis for the purpose of [^14^C]sugar identification was conducted on the insoluble material (fraction 2 [see Fig. S4 in the supplemental material]) that contains nascent PG with newly synthesized AG material. Hydrolysates were then reduced and per-*O*-acetylated before analysis via silica TLC. Analysis of per-*O-*acetylated alditol acetates from this insoluble fraction revealed a major band that migrated to the position of per-*O-*acetylated galactitol (Fig. 5B, lane 2). Other bands that correspond to per-*O-*acetylated glucitol and per-*O-*acetylated N-acetyl glucitol were also identified (Fig. 5B, lane 2). However, we observed no statistically significant variation in the intensity of these bands across all samples and repeats and attribute this phenomenon to metabolism of UDP-[^14^C]Gal into other sugars under these reaction conditions (Fig. 5B, lane 2), and this phenomenon has also been previously reported (12). Overall, this background activity [^14^C]Gal incorporation can be attributed to endogenous ^Ms^Lcp1 present in the *M. smegmatis* membrane preparation used in the assay mixture (Fig. 5B, lane 2). Due to the essentiality of *^Ms^*lcp1, we were unable to culture *M. smegmatis* depleted of ^Ms^Lcp1 in order to derive membranes free from ^Ms^Lcp1 activity. Therefore, we adopted a strategy whereby we assayed for Lcp1 ligase activity over and above endogenous background activity (Fig. 5B, lane 2). The addition of recombinant ^Mtb^Lcp1 to reaction mixtures resulted in a concomitant increase in band density that corresponds to per-*O-*acetylated galactitol (Fig. 5B, lanes 3 and 4), which is also quantified by densitometric analysis. However, in control reaction mixtures where PPG is removed from the assay mixture, we observed no significant incorporation of [^14^C]Gal*f*-labeled material, as manifested by the disappearance of a band corresponding to per-*O-*acetylated galactitol (Fig. 5B, lane 5). Taken together, these data clearly demonstrate two major findings. First, we are able to directly monitor the ligation of [^14^C]Gal*f* cell wall material to PG. Second, recombinant ^Mtb^Lcp1 is capable of using decaprenyl-P-P-[^14^C]Gal*f_n_* as a substrate for subsequent attachment to PG.

## DISCUSSION

After conducting a bioinformatics analysis of organisms belonging to the *Actinomycetales*, we identified ^Mtb^Lcp1 (Rv3267) as the primary putative LCP homolog in *M. tuberculosis*, sharing 20% identity with Cps2A from *S. pneumoniae* (see Fig. S1 in the supplemental material)*.* Since *lcp1* is positioned immediately upstream of two genes involved in linker unit biosynthesis, in the form of *wbbL1* and *rmlD* (Fig. 1A), we decided to investigate the *M. smegmatis* lcp1 ortholog (MSMEG_1824) as a model system to study the molecular genetics of this gene in order to assess its essentiality and to confirm its role as a primary LCP protein involved in mycobacterial cell wall assembly.

Mycobacterial AG is a highly branched heteropolysaccharide that serves to attach PG to the outer “mycomembrane,” and the biosynthesis of this essential cell wall component has been extensively studied (reviewed in reference 9). Since we have previously used *M. smegmatis* to successfully study the molecular genetics of mycobacterial cell wall biosynthesis (22, 24), we attempted to generate a clean deletion of *^Ms^*lcp1 from *M. smegmatis*. However, due to the essential nature of *^Ms^*lcp1 in attaching AG to PG in the cell envelope, we anticipated that a direct deletion of *^Ms^*lcp1 would result in a nonviable, lethal strain, and our hypothesis was justified by our inability to generate a null mutant. The essentiality of *^Ms^*lcp1 was confirmed when we performed a CESTET essentiality experiment (Fig. 3) (22). Generating null mutants of genes in mycobacteria that are involved in essential aspects of cell envelope biosynthesis is technically challenging and can be attributed to corresponding defects in the cell wall that produce nonviable phenotypes. The *M. smegmatis* Δ*aftC* mutant represents a strain of mycobacteria for which we have succeeded in generating a significant cell wall defect via genetic manipulation (24). Due to the complete loss of α(1→3)-arabinosyltransferase activity, the *M. smegmatis* Δ*aftC* mutant produces a significantly altered AG but, crucially, still connects PG to the outer (albeit reduced) mycolate layer (24). Unsuccessful attempts by our laboratory to generate a direct knockout (KO) of any other gene(s) involved in cell wall biosynthesis upstream of this event are due to the formation of nonviable mutants which are completely unculturable. Given the essential nature of the mycobacterial cell wall toward cell viability, the generation of conditional mutants followed by phenotypic characterization via the CESTET procedure is an extremely useful tool to confirm gene essentiality. Furthermore, *M. smegmatis* encodes three additional Lcp orthologs in the forms of the products of *MSMEG_0107*, *MSMEG_6421*, and *MSMEG_5775*, none of which can complement the function of MSMEG_1824 KO. In *M. tuberculosis*, two additional Lcp orthologs are encoded by *Rv3484* and *Rv0822c*. In this regard, the function of these remaining *lcp* genes requires further investigation.

**FIG 6:**
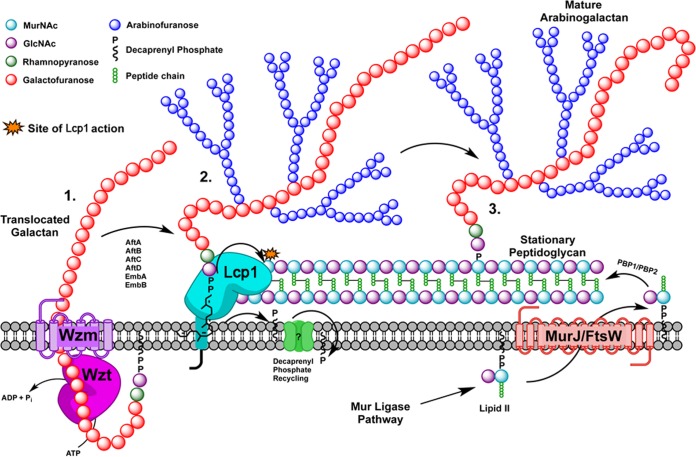
Schematic diagram of the latter stages of mycobacterial cell wall biosynthesis. (1) Translocation of galactan polymer across plasma membrane. Lcp1 then recognizes and binds the linker unit (i.e., represented by compounds 2 and 3). (2) Phosphotransferase activity links AG to the 6′-OH of muramyl residues, releasing decaprenyl-1-monophosphate for recycling. (3) Newly bound AG is released by Lcp1 to go on to form mature mAGP. Additional synthesis of new PG, requiring AG addition by Lcp1, takes place simultaneously.

The presence of a hydrophobic channel within the ^Mtb^Lcp1 homology model is consistent with similar cavities found within LCP proteins from *B. subtilis* and *S. pneumoniae* (13, 15) (Fig. 1C). Indeed, biochemical analysis of recombinant ^Mtb^Lcp1 clearly demonstrates that decaprenyl-1-monophosphate copurifies and is likely to be bound within the hydrophobic cavity of the protein (Fig. 2). In order to investigate the biochemical function of ^Mtb^Lcp1, we chemically synthesized a panel of four ligands that represent chemical structures found within the mycobacterial AG proximal to the linker unit and used intrinsic ITF to study the ligand binding properties of these ligands against ^Mtb^Lcp1. We observed a ligand binding relationship that reached saturation with a defined *B*_max_ for each of the compounds 1 to 3, all of which display a binding affinity in the low-micromolar range. Compound 1 was synthesized to mimic the l-Rha-α(1→3)-d-GlcNAc disaccharide moiety of the linker unit, and we recorded a *K_D_* of 58.71 µM. However, our binding data clearly demonstrate that the ligand with highest affinity for ^Mtb^Lcp1 is the tetrasaccharide Gal*f*_2_-Rha-GlcNAc-*O*-C_8_ (compound 2) with a *K_D_* of 5.13 µM. Interestingly, extension of this molecule with an additional Gal*f* residue (compound 3) caused a subtle 4-fold decrease in binding affinity (Fig. 4B). For technical reasons, we were unable to synthesize Gal*f*-Rha-GlcNAc-*O*-C_8_ and therefore were unable to study the binding of a ligand that represents the linker unit with only a single Gal*f* extension. In a control experiment, we investigated the ligand binding properties of Gal*f*-β(1→4)-l-Rha*p* (compound 4) toward ^Mtb^Lcp1. This compound displayed no recordable affinity toward the protein (over changes in background fluorescence), which suggests that the presence of d-GlcNAc in the linker unit is vital for biophysical association between protein and ligand. However, since ^Mtb^Lcp1 is able to bind both the linker unit (compound 1) and the linker unit with 2 to 3 Gal*f* residues (compounds 2 and 3, respectively), our data suggest that ^Mtb^Lcp1 might catalyze the phosphotransfer and ligation of AG to PG upon encountering a true physiological substrate that contains a decaprenyl-pyrophosphate-linker unit with at least one (if not two) Gal*f* residue at the nonreducing end of the glycan chain (Fig. 6).

We extended our investigation toward the development of a cell-free functional assay to measure the ligation of AG to PG in the presence of ^Mtb^Lcp1. Chemical analysis of a reaction mixture that contained ^14^C-radiolabeled AG supplemented with nascent PG clearly shows that ^Mtb^Lcp1 is capable of ligating both macromolecules in a titratably dependent manner (Fig. 5). However, because this assay requires the use of mycobacterial membranes that contain endogenous AG-PG ligase activity, an alternative explanation is that recombinant ^Mtb^Lcp1 could indirectly activate this residual membrane activity. It has been previously reported that ligation of AG to PG in mycobacteria requires concomitant synthesis of both cell wall polymers (10, 12); however, until now, the enzyme responsible for this activity has not been reported. This study now provides compelling genetic and biochemical evidence that ^Mtb^Lcp1 is indeed the enzyme that reconciles two major mycobacterial cell wall biosynthetic pathways by forming a covalent phosphodiester between PG and AG (Fig. 6). The discovery of ^Mtb^Lcp1 sheds new light on a key reaction involved during late-stage cell wall assembly which is likely to be critical for all members of the *Actinomycetales* harboring AG attached to PG via a phosphodiester linker unit.

In *S. aureus*, the first two genes of the WTA pathway (*tagO* and *tagA*) are nonessential and can be deleted without retarding growth of both mutant strains (33, 34). However, deletion of any of the subsequent WTA-synthesizing enzymes (TagBDFGH or TarIJL) produces a lethal phenotype, unless each corresponding mutant also contains an inactivation in *tagO* or *tagA* (34). A proposed hypothesis to explain this synthetic viable phenotype relates to the limited availability of the bactoprenol lipid carrier in the cytoplasmic membrane, which is required for not only WTA biosynthesis but also lipid II biosynthesis, which ultimately leads to PG assembly (34). Furthermore, similar effects have been demonstrated in *S. aureus* by chemical means, whereby targocil, an inhibitor of late-stage WTA biosynthesis, causes an accumulation of undecaprenyl-linked intermediates, thus preventing recycling of the undecaprenyl-phosphate lipid carrier, causing synthetic lethality (35). This phenomenon most likely reflects the phenotypic consequence of an *^Ms^*lcp1 deletion in *M. smegmatis*. Upon acetamide withdrawal from growing cultures of *M. smegmatis* Δ^Ms^lcp1 pMV306-ACET-*^Ms^*lcp1, expression of *^Ms^*lcp1 diminishes, which will likely result in two major effects: (i) loss of AG-PG ligase activity and (ii) a concomitant increase in the decaprenyl-pyrophosphoryl-linked galactan intermediate(s). While the former effect induces a devastating cell wall lesion, the latter effect will cause a dramatic reduction in available decaprenyl monophosphate that is also required for PG biosynthesis, via loss of lipid II. Furthermore, *Actinomycetales* such as *M. tuberculosis* and *C. glutamicum* also use decaprenyl phosphate in the production of decaprenyl-phosphoarabinose (DPA) and decaprenyl-phosphomannose (DPM), which is directed toward the formation of d-arabinan and lipomannan (which is the precursor to lipoarabinomannan [LAM]), respectively (36, 37). Benzothiazinones (BTZs) are a class of compounds that are particularly potent inhibitors of mycobacterial growth. BTZs target DprE1, which is an oxidoreductase involved in the epimerization of decaprenyl-phosphoribose (DPR) to decaprenyl-phosphoarabinose (DPA), thus arresting d-arabinan biosynthesis (38, 39). However, it has recently been shown that BTZ’s potent mode of action is attributed to the blocking of decaprenyl monophosphate recycling, a process which is required to allow cell wall biosynthesis to function properly (40). In this regard, Lcp1 is a potential drug target, since its inhibition will affect late-stage cell wall maturation and reduce the availability of decaprenyl monophosphate, leading to synthetic lethality.

## MATERIALS AND METHODS

### Homology modeling and sequence alignment.

The amino acid sequence of *M. tuberculosis* Rv3267 (^Mtb^Lcp1, Swiss-Prot ID P96872_MYCTU) was submitted for three-dimensional (3D) structure prediction using the I-TASSER server (19, 20), and the conformation of the secondary structural features for the resultant ^Mtb^Lcp1 model was predicted using pdbsum (41). The homology model of ^Mtb^Lcp1 was analyzed in comparison to the Cps2A structures from *S. pneumoniae* (PDB ID 2XXP) (13).

### Chemicals, reagents, and enzymes.

All chemicals and solvents were from Sigma-Aldrich (Dorset, United Kingdom), Bio-Rad (CA, USA), and Fisher Chemicals (United Kingdom) unless otherwise stated and were of analytical grade or equivalent. Plasmids were propagated during cloning in *Escherichia coli* Top10 cells (Invitrogen). All restriction enzymes and Phusion DNA polymerase enzyme were sourced from New England Biolabs. The Bioline quick ligation kit was used to perform ligation reactions. Oligonucleotides were from MWG Biotech Ltd., and PCR fragments were purified using the QIAquick gel extraction kit (Qiagen). Plasmid DNA was purified using the QIAprep purification kit (Qiagen). The preparation of oligosaccharides 1 to 3 using methods previously reported (42) is described in Text S1 in the supplemental material. Disaccharide 4 was prepared previously (43).

### Bacterial strains and growth conditions.

*E. coli* Top10 cells were routinely grown in Luria-Bertani broth or Luria-Bertani agar (LB; Difco) at 37°C. *E. coli* BL21(DE3) cells were grown in Terrific Broth (Difco) at 37°C. *M. smegmatis* mc^2^155 was grown in tryptic soy broth (TSB; Difco) containing Tween 80 supplemented with the appropriate antibiotic(s) (25 µg/ml kanamycin [Kan] and/or 100 µg/ml hygromycin).

### Construction of recombinant plasmids.

For generation of the ^Mtb^Lcp1 expression vector, a 1,389-bp region of Rv3267 was cloned from *M. tuberculosis* H37Rv genomic DNA using the following primer pairs (restriction sites underlined): Rv3267FW (5′-GATCGATCCATATGCGGTCGTTCGAAGACGGCATCTTCCAC-3′) and Rv3267DN (5′-GATCGATCAAGCTTTCAGTTGATGCACTCCGGCGCGTCGGAG-3′). The PCR product was ligated into pET28b vector and cut with NdeI and HindIII, yielding pET28b-*^Mtb^*lcp1. The plasmid was subsequently sequenced to check for construct integrity (Eurofins MWG).

For generating an integrative vector containing *MSMEG1824*, the open reading frame (ORF) was amplified from *M. smegmatis* mc^2^155 genomic DNA using the primers MS1824FW (5′-GATCGATCGATATCTTGATCAGGTCCATTGCTGTGGCCGCA-3′) and MS1824DN (5′-GATCGATCAAGCTTTCAGTTCACGCACTGCGGGTCGTTGGC-3′). The DNA amplicon was digested with HindIII and EcoRV and subsequently cloned into pMV306 together with the P_acetamidase_ promoter region from pSD26 as described previously (22), thus yielding pMV306-ACET-*MSMEG1824* (pMV306-ACET-^Ms^lcp1). The plasmid was subsequently sequenced to check for construct integrity (Eurofins MWG).

For generation of the Δ*^Ms^*lcp1 phasmid, approximately 1-kb sequences of the upstream and downstream regions of *MSMEG1824* were PCR amplified from *M. smegmatis* mc^2^155 genomic DNA with the primer pairs MS1824LL (5′-TTTTTTTTCCATAAATTGGCGACCACCAGGGGGCGGGCATCGTCCA-3′) and MS1824LR (5′-TTTTTTTTCCATTTCTTGGACCGGGGCAGGCGGCATGGTCGGGCGC-3′) and MS1824RL (5′-TTTTTTTTCCATAGATTGGACGACGCCACCGGTGAACGCATCGAGC-3′) and MS1824RR (5′-TTTTTTTTCCATCTTTTGGGCCGACGACAGGCTGCTCGACGACTAC-3′), respectively (all primers had Van91I recognition sites incorporated at the 5′ end). The PCR fragments were digested with Van91I and directly cloned into Van91I-digested p0004S (gift from T. Hsu and W. R. Jacobs, Jr., Albert Einstein College of Medicine, NY). Recombinant plasmids obtained after transforming *E. coli* Top10 cells were digested with Van91I for confirmation and sequenced. One plasmid, pΔ*MSMEG1824*, was linearized by PacI digestion and packaged into the temperature-sensitive mycobacteriophage phAE159, as previously described (44), to yield phasmid DNA of the knockout phage phΔ*MSMEG1824* (phΔ^Ms^lcp1).

### Generation of the Δ*^Ms^lcp1* conditional mutant.

The *M. smegmatis* Δ*^Ms^*lcp1 conditional mutant was generated using the CESTET procedure (22). Briefly, a merodiploid strain was first generated by introducing pMV306-ACET-^Ms^lcp1 by electroporation into *M. smegmatis* mc^2^155 (22). The merodiploid strain *M. smegmatis* mc^2^155::pMV306-ACET-^Ms^lcp1 was then subjected to specialized transduction as previously described (22) using a temperature-sensitive, recombinant phage, phΔ^Ms^lcp1, designed to replace *MSMEG1824* with a hygromycin resistance marker. Transductants were selected at the nonpermissive temperature of 37°C on selective plates containing 25 µg/ml kanamycin, 100 µg/ml hygromycin B, and 0.2% (wt/vol) acetamide. Allelic exchange in hygromycin-resistant transductants was confirmed by PCR and Southern blotting.

### Conditional depletion of the Δ*^Ms^lcp1* conditional mutant.

The Δ*^Ms^*lcp1 conditional mutant was grown in tryptic soy broth (TSB; Difco) containing 0.05% Tween 80, 25 µg/ml kanamycin, 100 µg/ml hygromycin B, and 0.2% (wt/vol) acetamide and subsequently passaged twice in medium with and without acetamide. Ten-milliliter cultures were allowed to grow for a further 56 h at 37°C with shaking. Ten-microliter samples were taken at 8-h intervals and plated on TBS agar plates supplemented with 25 µg/ml kanamycin and 100 µg/ml hygromycin B with or without 0.2% (wt/vol) acetamide to measure CFU.

### Expression and purification of ^Mtb^Lcp1.

The plasmid pET28b-*^Mtb^*lcp1 was transformed into *E. coli* BL21(DE3) cells, spread onto LB agar plates containing 50 mg/ml Kan, and incubated at 37°C for 18 h. Single colonies were selected to inoculate 5 ml LB broth containing 50 µg/ml Kan and were incubated at 37°C overnight. The overnight cultures were then used to inoculate 1 liter Terrific Broth containing 50 mg/ml Kan, which was incubated (37°C, 180 rpm) until an optical density at 600 nm (OD_600_) of 0.5 was reached. Protein production was then induced by the addition of 1 mM IPTG (isopropyl-β-d-thiogalactopyranoside; 12 h at 16°C). Cells were harvested via centrifugation at 6,000 × *g* for 15 min (4°C). Supernatant was discarded, and cells were washed in 30 ml phosphate-buffered saline (PBS). Pellets were stored at −20°C until further use. A pellet was defrosted and then resuspended in 30 ml lysis buffer (50 mM KH_2_PO_4_ [pH 7.9], 300 mM NaCl, 20 mM imidazole), including a proteinase inhibitor tablet (Roche). The mixture was subsequently sonicated using a Soniprep 150 ultrasonic disintegrator (MSE) (30 s on, 30 s off, 10 cycles). The solution was then centrifuged at 27,000 × *g* for 40 min (4°C), and the supernatant was retained (clarified lysate). Protein was loaded onto a HisTrap 5-ml chelating HP column (GE Healthcare), charged with 0.1 M NiCl_2_, equilibrated in lysis buffer, and eluted via a stepwise 50 to 500 mM imidazole concentration gradient (in lysis buffer). Fractions were analyzed by 12% SDS-PAGE to check for purity, and those fractions containing purified protein were dialyzed into dialysis buffer (25 mM Tris-HCl [pH 7.9], 10 mM NaCl, and 10% glycerol). Protein concentration was measured using a Bradford assay.

### Extraction of decaprenyl-1-monophosphate from ^Mtb^Lcp1.

Two milliliters of recombinant ^Mtb^Lcp1 (2 mg/ml dissolved in dialysis buffer) was combined with 2 ml CHCl_3_ and 1 ml of CH_3_OH and heated at 60°C for 2 h with agitation. After centrifugation at 5,000 × *g* for 10 min, the lower organic fraction was recovered and dried under nitrogen. The resulting extract was resuspended in CHCl_3_-CH_3_OH (100 µl, 2:1, vol/vol) and analyzed by electrospray mass spectrometry (ES-MS) in the negative mode on a Micromass LCT mass spectrometer. An identical extraction was also carried out on 2 mg/ml of EmbC^CT^ (21), which was used as a negative control.

### Ligand binding experiments.

Intrinsic tryptophan fluorescence (ITF) ligand binding experiments of ^Mtb^Lcp1 with saccharide ligands were conducted on a Hitachi F7000 fluorescence spectrophotometer with the temperature thermostatically regulated to 25°C. ^Mtb^Lcp1 was diluted to 0.026 µM with dialysis buffer, and fluorescence spectra were measured at an excitation wavelength of 280 nm and emission wavelength of 300 to 400 nm with an excitation and emission slit width of 5 nm using a 500-µl crystal cuvette. ITF maximum (*F*_emission_^max^) was recorded at λ = 341 nm, providing a basal *F*_emission_ coordinate for the collection of subsequent ITF data*.* Increasing concentrations of ligands representing the linker unit (compounds 1 to 4) were added to the cuvette. The change in fluorescence emission (Δ*F*_emission_) was calculated by subtracting *F*_emission_ (recorded 2 min after each ligand addition) from *F*_emission_^max^*.* Changes of background fluorescence observed upon addition of equivalent volumes of dialysis buffer (the solvent in which compounds 1 to 4 were dissolved) were subtracted from the overall change in fluorescence, and the data were then plotted against ligand concentration, [L] (3 independent biological experiments). A plot of Δ*F*_emission_ at λ = 341 nm versus [L] was fitted to a one-site specific binding equation using GraphPad Prism 5 software: Δ*F*_emission_ = *F*_max_ × [L]/(*K_D_* + [L]).

### Preparation of nascent mycobacterial peptidoglycan.

One liter of *M. smegmatis* mc^2^155 was cultured in TSB as described above and harvested by centrifugation. Pelleted *M. smegmatis* cells were resuspended in H_2_O before addition of CHCl_3_-CH_3_OH (1:1, vol/vol) to create CHCl_3_-CH_3_OH-H_2_O (10:10:3, vol/vol/vol) single phase. The suspension was centrifuged at 3,300 × *g* for 15 min, the resulting supernatant was discarded, and the pellet was resuspended in 30 to 40 ml H_2_O. The cell suspension was sonicated (60 s on, 90 s off, 15 cycles) before addition of 1% (vol/vol) Triton X-100. The cell suspension was incubated at room temperature for 16 h and then centrifuged at 3,300 × *g* for 15 min. The crude insoluble material from the above extraction was extracted three times with 2% SDS in PBS at 95°C for 1 h; washed with water, 80% (vol/vol) acetone in water, and acetone; and finally lyophilized to yield a highly purified cell wall mAGP preparation (45). Mycolic acids were removed from mAGP by treatment with 0.5% (wt/vol) KOH in methanol at 37°C for 4 days. The treated mAGP was collected by centrifugation at 27,000 × *g* for 20 min and then washed repeatedly with methanol and recentrifuged at 27,000 × *g*, and the pellet was recovered. The mycolic acids were then extracted from the treated mAGP using diethyl ether and recentrifuged at 27,000 × *g*, and the pellet (AGP) and soluble mycolic acid methyl esters were recovered in the diethyl ether supernatant. The extraction process using diethyl ether was repeated three times. The AGP complex was hydrolyzed with 0.2 M H_2_SO_4_ at 85°C for 30 min and neutralized with BaCO_3_ (32). The supernatant (which contained solubilized arabinogalactan [AG]) was removed via centrifugation at 27,000 × *g* for 30 min. The residual pellet (containing the highly purified peptidoglycan [PPG] fraction) was finally extensively washed with water, freeze-dried, and stored at −20°C.

### Functional biochemical assay for ^Mtb^Lcp1.

Membranes (containing membrane-bound enzymes involved in lipid-linked cell wall biosynthetic processes) and P60 (a Percoll-derived cell-free fraction rich in carbohydrate and enzymes associated with cell wall processes as described in reference 46) fractions from *M. smegmatis* were prepared as described previously (24, 26, 46) and resuspended in buffer A (50 mM morpholinepropanesulfonic acid [MOPS; pH 7.9] containing 5 mM β-mercaptoethanol and 10 mM MgCl_2_) to a final concentration of 15 mg/ml and 10 mg/ml for membrane and P60 fractions, respectively.

The basic assay mix consisted of buffer A, 0.5 mg membranes (33 µl), 0.5 mg of P60 (50 µl), 0.2 mM ATP, 0.2 mM NADH, 0.1 µCi of UDP-d-[U-^14^C]galactose (specific activity, 300 mCi/mmol; ARC Radiochemicals), and 1 mg PPG; the reaction was initiated with the addition of 0.1 mg/ml ^Mtb^Lcp1 (final volume of 200 µl); and the reaction mixture was incubated for 17 h at 37°C. Reactions were quenched by the addition of 1,333 µl of CHCl_3_-CH_3_OH (1:1, vol/vol). After mixing and centrifugation at 27,000 × *g* for 15 min at 4°C, the supernatant was removed and dried under nitrogen and the pellet was retained for further processing. The dried supernatant was resuspended into 2 ml of CHCl_3_-H_2_O (1:1, vol/vol), and the lower organic phase (fraction 1) was removed and dried under nitrogen. Material in fraction 1 was subjected to TLC using CHCl_3_-CH_2_OH-H_2_O-NH_4_OH (65:25:3.6:0.5, vol/vol/vol/vol) on aluminum-backed silica gel plates (5735 silica gel 60F254; Merck), and products were visualized by autoradiography, exposing the TLCs to X-ray film (Kodak X-Omat). The pellet from the previous step was resuspended into 1 ml H_2_O-C_2_H_5_OH-(C_2_H_5_)_2_-pyridine-NH_4_OH (15:15:5:1:0.017, vol/vol/vol/vol/vol) and left at room temperature to be extracted for 2 h. Samples were again centrifuged at 27,000 × *g* for 15 min, and supernatant was discarded. The remaining pellet (fraction 2) was resuspended in 200 µl H_2_O. All fractions were quantified by liquid scintillation counting using 10% of the labeled material and 5 ml of EcoScintA (National Diagnostics, Atlanta, GA).

Material in fraction 2 was hydrolyzed in 2 M trifluoroacetic acid (TFA) and reduced with NaBH_4_, and the resultant alditols were per-*O-*acetylated as described previously (47). The resulting ^14^C-labeled per-*O-*acetylated alditol acetate derivatives were analyzed by TLC using ethyl acetate-hexane (4:6, vol/vol) on aluminum-backed silica gel plates (5735 silica gel 60F254; Merck), and products were visualized by autoradiography, exposing the TLCs to X-ray film (Kodak X-Omat) and comparing them to known standards. All experiments were performed in triplicate as biological replicates.

## SUPPLEMENTAL MATERIAL

Text S1  Supplemental methods. Download Text S1, PDF file, 0.2 MB

Figure S1  Sequence alignment of Lcp1 orthologues from *M. tuberculosis*, *M. smegmatis*, and *C. glutamicum* and LCP homologs from *B. subtilis* and *S. pneumoniae*. Amino acid sequences were aligned using ClustalW and rendered with EsPRIPT. Secondary structure information was obtained from PDB coordinates 2XXP. Download Figure S1, PDF file, 2.6 MB

Figure S2  Reaction scheme for the synthesis of octyl α-l-rhamnopyranosyl-(1→3)-2-acetamido-2-deoxy-α-d-glucopyranoside (compound 1). Download Figure S2, PDF file, 0.2 MB

Figure S3  Reaction scheme for the synthesis of octyl-β-d-galactofuranosyl-(1→5)-β-d-galactofuranosyl-(1→4)-α-l-rhamnopyranosyl-(1→3)-2-acetamido-2-deoxy-α-d-glucopyranoside (compound 2) and octyl-β-d-galactofuranosyl-(1→6)-β-d-galactofuranosyl-(1→5)-β-d-galactofuranosyl-(1→4)-α-l-rhamnopyranosyl-(1→3)-2-acetamido-2-deoxy-α-d-glucopyranoside (compound 3). Download Figure S3, PDF file, 0.8 MB

Figure S4  Flow chart tracking radioactivity incorporated from UDP-[^14^C]Gal*p* through each of the analytical steps leading to TLC analysis (Fig. 5). Download Figure S4, PDF file, 0.4 MB
