# Supplementary material for: Lcp1 Is a Phosphotransferase Responsible for Ligating Arabinogalactan to Peptidoglycan in Mycobacterium tuberculosis
Source: mBio. 2016 Aug 2;7(4):e00972-16. doi: 10.1128/mBio.00972-16 (PMC4981717; doi:10.1128/mBio.00972-16)
Supplement: Text S1 — Supplemental methods. Download [file mbo004162922s1.pdf]

Supplementary Information

Lcp1 is a phosphotransferase responsible for ligating arabinogalactan to peptidoglycan in *Mycobacterium tuberculosis*

James Harrison, Georgina Lloyd, Maju Joe, Todd L. Lowary, Edward Reynolds, Hannah Walters-Morgan, Andrew Lovering, Gurdyal S. Besra, Luke J. Alderwick

Chemical Synthesis

It was decided to substitute the pyrophosphate linked polyprenyl of the endogenous acceptor with an *O*-alkyl group, because pyrophophate-linked polyprenols are difficult to use as acceptors as they are unstable. An octyl glycoside was chosen as a replacement because these have previously been used successfully as synthetic ligands and acceptors (1). Disaccharides containing L-Rha-α(1→3)-D-GlcNAc have been synthesised previously (2, 3) using a thioglycoside donor, which activated *in situ* from *N*-iodosuccinimide (NIS) and triflic (TfOH) acid (4). The scheme used this coupling method and is shown in Scheme S2. To synthesize **1**, thioglycoside **6** (5) and alcohol **5** (6) were coupled using *N*-iodosuccinimide (NIS) and trifluoromethanesulphonic (triflic) acid leading to octyl 2,3,4-tri-*O*-acetyl-α-L-rhamnopyranosyl-(1→3)-2-acetamido-4,6-*O*-benzylidene-2-deoxy-α-D-glucopyranoside **(7)** (Scheme S2) in 51% yield. The heteronuclear one-bond coupling constant ($^{1}J_{C,H}$) for the anomeric C atom (172 Hz) unambiguously established that the compound had the required α-*rhamno* configuration (7). Deprotection of **7** using hydrogenolysis of the benzylidene acetyl followed by cleavage of the acetate esters with methanolic ammonia afforded the final product, α-L-rhamnopyranosyl-(1→3)-2-acetamido-2-deoxy-α-D-glucopyranoside **(1)** in 80% yield over the two steps.

The preparation of **2** and **3** followed a common synthetic route as illustrated in Scheme S3 starting from the previously reported disaccharide **8** and two thioglycosides **9** and **10**, which were prepared following established protocols (8). Glycosylation of disaccharide **8** with thioglycoside **9** using NIS and silver triflate provided trisaccharide **11**, which was the then treated with hydrazine acetate affording alcohol **12** in 87% overall yield from **8**. Subsequent glycosylation of **12** with thioglycoside **10**, again promoted by NIS and silver triflate, gave an 89% yield of tetrasaccharide **13**. A portion of this intermediate

was deprotected by hydrolysis of the acetal protecting groups and then hydrolysis of the esters under Zemplen conditions to afford **2** in 93% yield. Alternatively, another portion of **13** was treated with hydrazine acetate affording tetrasaccharide alcohol **14** in 97% yield. Glycosylation with **9** afforded **15** (81% yield), which was then deprotected under the same conditions used for **2**, leading to the formation of **3** in 91% overall yield. In all glycosylation reactions, the stereochemistry of the newly introduced galactofuranosyl residue could be determined by ^{1}H NMR and ^{13}C NMR spectroscopy. In particular, the $^{3}J_{H1,H2}$ of the galactofuranosyl residue was <2 Hz and the C-1 chemical shift was between 105 and 110 ppm (9).

Supplementary Figure Legends

Figure S1. Sequence alignment of Lcp1 orthologues from *M. tuberculosis, M. smegmatis, C. glutamicum* and LCP homologues from *B. subtilis* and *S. pneumoniae*. Amino acid sequences were aligned using ClustalW and rendered with EsPRIPT. Secondary structure information was obtained from PDB coordinates 2xxp.

Figure S2. Reaction scheme for the synthesis of octyl α-L-rhamnopyranosyl-(1→3)-2-acetamido-2-deoxy-α-D-glucopyranoside (**1**).

Figure S3. Reaction scheme for the synthesis of octyl β-D-galactofuranosyl-(1→5)-β-D-galactofuranosyl-(1→4)-α-L-rhamnopyranosyl-(1→3)-2-acetamido-2-deoxy-α-D-glucopyranoside **(2)** and octyl β-D-galactofuranosyl-(1→6)-β-D-galactofuranosyl-(1→5)-β-D-galactofuranosyl-(1→4)-α-L-rhamnopyranosyl-(1→3)-2-acetamido-2-deoxy-α-D-glucopyranoside **(3)**.

Figure S4. Flow chart tracking radioactivity incorporated from UDP-[^{14}C[Gal*p* through each of the analytical steps leading to TLC analysis (Figure 5).

Supplementary Methods

Synthesis of 1–3.

General Methods. Reactions were carried out in oven-dried glassware. All reagents used were purchased from commercial sources and were used without further purification unless noted. Reaction solvents were purified by successive passage through columns of alumina and copper under nitrogen. Unless stated otherwise, all reactions were carried out at room temperature under a positive pressure of argon and were monitored by TLC on Silica Gel 60 F_{254} (0.25 mm, E. Merck). Spots were detected under UV light or by charring with acidified *p*–anisaldehyde solution in EtOH. Unless otherwise indicated, all column chromatography was performed on Silica Gel (40–60 μM). The ratio between silica gel and crude product ranged from 100 to 50:1 (w/w). Optical rotations were measured at 22 ± 2 °C and are in units of deg•mL(dm•g)$^{-1}$. ^{1}H NMR spectra were recorded at 300 or 500 MHz, and chemical shifts were referenced to TMS (0.0, $CDCl_3$) or CD_3OD (3.30, CD_3OD). ^{13}C NMR spectra were recorded at 75 or 125 MHz, and ^{13}C chemical shifts were referenced to internal $CDCl_3$ (77.06 ppm, $CDCl_3$), or CD_3OD (48.9, CD_3OD). Organic solutions were concentrated under vacuum at < 40 °C. Electrospray mass spectra were recorded on samples suspended in mixtures of THF with MeOH and added NaCl. MALDI mass spectrometry was performed on a Voyager Elite time-of-flight spectrometer on samples suspended in 2,5-dihydroxy-benzoic acid or *trans*-3-indoleacrylic acid using the delayed-extraction mode and positive-ion detection.

Octyl α-L-rhamnopyranosyl-(1→3)-2-acetamido-2-deoxy-α-D-glucopyranoside (1) . Disaccharide **7** (110 mg, 0.159 mmol) was dissolved in acetic acid–H_2O (5 mL, 80:20) and subjected to hydrogenolysis using palladium on carbon (10%, 15 mg). The reaction mixture

was stirred overnight and filtered through Celite and evaporated. The residue was co-evaporated thrice with toluene and dissolved in ammonia–MeOH (15 mL, 2.0M) and stirred for 20 h. The solution was evaporated to dryness. Flash column chromatography (0–15% MeOH in $CHCl_3$) of the residue gave **1** as a white powder (76 mg, 80%). M.P. 120–121°C, $[\alpha]_D$ –13.4 (*c* 1, $CHCl_3$). δ_H (300 MHz; CD_3OD) 4.91 (s, 1 H), 4.74 (d, 1 H, *J* = 2.8 Hz, H-1), 3.51–4.15 (10 H), 2.05 (s, 3 H, NHCOCH_3), 1.51–1.60 (2 H, m), 1.25–1.46 (m, 13 H), 0.86 (m, 3 H); δ_C (75 MHz; CD_3OD) 173.4, 101.2, 97.5, 79.7, 72.3, 72.0, 71.0, 70.4, 69.0, 68.1, 68.1, 60.5, 53.4, 31.9, 29.4, 29.2, 29.1, 26.0, 22.7, 22.3, 16.8, 13.9 (C-a). *m/z* (EI) 480.2831 (M^+ + H. $C_{22}H_{42}NO_{10}$ requires 480.2809).

Octyl β-D-galactofuranosyl-(1→5)-β-D-galactofuranosyl-(1→4)-α-L-rhamnopyranosyl-(1→3)-2-acetamido-2-deoxy-α-D-glucopyranoside (2).

Tetrasaccharide **13** (0.2 g, 0.12 mmol) was dissolved in 4:1 acetic acid–H_2O (8 mL) and heated at 75–77 °C for 5 h. The solvent was then evaporated under vacuum and the residue was dissolved in CH_2Cl_2–MeOH (7:3, 5 mL) followed by the dropwise addition of $NaOMe_3$ in MeOH (0.1M, enough to maintain p*H* of the solution around 8). More MeOH was added periodically as the reaction progressed to keep the solution homogenous. The reaction mixture was then stirred at room temperature for 16 h and was neutralized by the addition of Amberlyst-15 (H+) cation exchange resin. The solution was filtered and the filtrate was concentrated to give a syrupy residue. This crude product was then dissolved in H_2O (0.5 mL) and was purified by C-18 column chromatography, (H_2O–MeOH, 0–75% MeOH). The fractions containing the product were combined, concentrated under vacuum, re-dissolved in deionized H_2O and lyophilized to obtain **2** (0.09 g, 93%) as an amorphous solid. The data for this compound matched with the data previously reported (8).

Octyl β-D-galactofuranosyl-(1→6)-β-D-galactofuranosyl-(1→5)-β-D-galactofuranosyl-(1→4)-α-L-rhamnopyranosyl-(1→3)-2-acetamido-2-deoxy-α-D-glucopyranoside (3).

Pentasaccharide **15** (0.2 g, 0.093 mmol) was dissolved in 4:1 acetic acid–H_2O (8 mL) and heated at 75–77 °C for 5 h. The solvent was then evaporated and the residue was dissolved in CH_2Cl_2–MeOH (7:3, 6 mL) followed by the dropwise addition of NaOMe in MeOH (0.1M, enough to maintain p*H* of the solution around 8). More MeOH was added periodically as the reaction progressed to keep the solution homogenous. The reaction mixture was then stirred at room temperature for 16 h and was neutralized by the addition of Amberlyst-15 (H+) cation exchange resin. The solution was filtered and the filtrate was concentrated to give a syrupy residue. This crude product was then dissolved in H_2O (0.5 mL) and was purified by C-18 column chromatography, (H_2O–MeOH, 0–75% MeOH). The fractions containing the product were combined, concentrated under vacuum, re-dissolved in deionized H_2O and lyophilized to obtain **3** (0.083 g, 91%) as an amorphous solid. The data for this compound matched with the data previously reported (8).

Octyl 2,3,4-tri-*O*-acetyl-α-L-rhamnopyranosyl-(1→3)-2-acetamido-4,6-*O*-benzylidene-2-deoxy-α-D-glucopyranoside (7) .

Methyl 2,3,4-tri-*O*-acetyl-1-thio α-L-rhamnopyranoside **(6)** (5) (500 mg, 1.56 mmol) and octyl 2-acetamido-2-deoxy-4,6-*O*-benzylidene-α-D-glucopyranoside **(5)** (6) (571 mg, 1.35 mmol) were dissolved in CH_2Cl_2 (80 mL); powdered 4Å molecular sieves (2.28 g) were added and the reaction mixture stirred for 1 h. *N*-iodosuccinimide (381 mg, 1 .69 mmol)

was then added. A satd solution of trifluoromethanesulfonic (trflic) acid in CH_2Cl_2 (1.05 mL, 0.15 mol equivalent) was added dropwise and the reaction mixture stirred in the dark. After 2 h, another 0.15 mol equivalent of the triflic acid in CH_2Cl_2 solution was added dropwise. A colour change of the reaction from pink to purple to brown was evident. After 7 h, the reaction was quenched by the addition of triethylamine (215 μL), filtered and diluted with CH_2Cl_2 and the solution was washed with sodium thiosulfate in NaOH (5 w/v, 0.5M), followed by aqueous sulphuric acid (1.0M), satd aqueous $NaHCO_3$, and brine before being dried over anhydrous $MgSO_4$ and evaporated to an oil. Flash column chromatography (0–50% EtOAc–toluene) afforded an off white solid that was recrystallised from ethanol–H_2O (50:50) to yield **7** as a white powder (480 mg, 51%). M.P. 151–152°C, $[\alpha]_D$ +4.2 (*c* 1, $CHCl_3$). δ_H (300 MHz) 7.30–7.45 (m, 5 H), 5.83 (d, 1 H, *J* = 8.8 Hz), 5.43 (s, 1 H), 4.95 (dd, 1 H, *J* = 4.0, 9.7 Hz), 4.90 (d, 1 H, *J* = 2.5 Hz), 4.88 (app t, 1 H, *J* = 9.7 Hz), 4.79 (d, 1 H, *J* = 3.5 Hz), 4.50 (br app t, 1 H, *J* = 2.5, 4.0 Hz), 4.20 (dd, 1 H, *J* = 4.2, 10.0 Hz), 4.07–4.15 (m, 1 H), 3.98 (br app t, 1 H, *J* = 9.1, 10.0 Hz), 3.73 (app dt, 1 H, *J* = 3.5, 9.6, 8.8 Hz), 3.66 (app t, 1 H, *J* = 9.6 Hz), 3.63 (dt, 1 H, *J* = 6.8, 9.9 Hz, octyl OCH_2), 3.53 (br app t, 1 H, *J* = 9.5, 8.5 Hz), 3.35 (dt, 1 H, *J* = 9.9, 6.7 Hz), 3.20 (dq, 1 H, *J* = 2.0, 8.7 Hz), 1.86, 1.94, 1.96, 1.98 (4 x 3H, 4 x s, 3 x $COCH_3$, 1 x $NHCOCH_3$), 1.51–1.60 (m, 2H octyl CH_2), 1.24–1.45 (m, 10 H, octyl CH_2), 1.09 (t, 3 H, octyl CH_3), 0.85 (d, 3 H, *J* = 2.0 Hz, H-6'), δ_C (75 MHz) 170.5, 170.3, 170.2, 170.0, 137.0–126.5, 102.2, 98.4 ($^1J_{C-,H}$ 172 Hz), 98.0, 80.4, 76.2, 71.6, 71.0, 69.2, 68.6, 68.5, 66.5, 63.3, 53.2, 32.0, 29.6, 29.5, 29.5, 26.4, 23.6, 22.8, 21.1, 20.9, 16.8, 14.3; Anal. Calcd. For $C_{35}H_{51}NO_{13}$: C: 60.6, H 7.4, N 2.1; found C 60.2, H 7.6, N 2.0. *m/z* (EI) 593.3193 (M^+–$(OCOCH_3)_2$+2H. $C_{31}H_{47}NO_{10}$ requires 593.3200).

Octyl 2,3,6-tri-*O*-benzoyl-β-D-galactofuranosyl-(1→4)-2,3-isopropylidene-α-L-rhamnopyranosyl-(1→3)-2-acetamido-4,6-*O*-benzylidene-2-deoxy-α-D-glucopyranoside (12).

To a solution of alcohol **8** (1.62 g, 2.67 mmol) and thioglycoside **9** (2.23 g, 3.2 mmol) in CH_2Cl_2 (80 mL) was added 4 Å molecular sieves (0.5 g) and the mixture was stirred for 30 min before cooling to 0 °C. NIS (0.86 g, 3.8 mmol) and silver trifluoromethane sulfonate (0.16 g, 0.6 mmol) were added and the mixture was continued to stir at 0 °C until the reaction was complete as determined by TLC. The reaction was then quenched by adding triethylamine until the pH of the solution was slightly basic (p*H* < 8). The reaction mixture was then quickly filtered into a solution of satd aq sodium thiosulfate (100 mL) and extracted with CH_2Cl_2. The organic layer was then washed with H_2O (2 × 70 mL), separated and dried over anhydrous Na_2SO_4. The organic layer was concentrated to give a syrupy residue that was filtered through a short silica column using 1:1 hexane–EtOAc as the eluent. The fractions containing **11** were combined and evaporated to give an oil that was dried under vacuum for 2 h before being carried forward. Crude **11** was then dissolved in CH_2Cl_2–MeOH (9:1, 60 mL) and then hydrazine acetate (0.36 g, 3.8 mmol), was added. The reaction was stirred for 1 h and then poured into H_2O and extracted with CH_2Cl_2. The organic layer was separated, dried with anhydrous Na_2SO_4 and concentrated to a syrupy residue that was purified by column chromatography to give **12** (2.5 g, 87% over two steps) as an amorphous solid. R_f 0.29 (55:45 hexane–EtOAc); $[\alpha]_D$ +4.7 (*c* 0.5, $CHCl_3$); ^1H NMR (500 MHz, $CDCl_3$, δ_H) 8.1–7.95 (m, 6 H), 7.62–7.50 (m, 3 H), 7.48–7.35 (m, 8 H), 7.20–7.03 (m, 3 H), 5.74 (s, 1H), 5.68 (d, 1 H, *J* = 10.0 Hz), 5.60 (d, 1 H, *J* = 4.9 Hz), 5.57 (s, 1 H), 5.53 (s, 1 H), 5.09 (s, 1H), 4.71 (d, 1 H, *J* = 3.7 Hz), 4.56 (dd, 1 H, *J* = 6.5, 10.8 Hz), 4.45–4.37 (m, 2 H), 4.37–4.23 (m, 3 H), 4.10 (d, 1H, *J* = 6.0 Hz), 3.98 (dd, 1 H, *J* = 9.6, 9.6 Hz), 3.95–3.80

(m, 2 H), 3.75 (dd, 1 H, *J* = 10.2 Hz), 3.74–3.3.67 (m, 1 H), 3.60 (dd, 1 H, *J* = 9.3 Hz), 3.52 (dd, 1 H, *J* = 7.6, 10.0 Hz), 3.40 (ddd, 1 H, *J* = 6.6, 9.7, 13.3 Hz), 2.66 (d, 1 H, *J* = 8.9 Hz), 2.03 (s, 3 H), 1.64–1.58 (m, 3 H), 1.52 (s, 3 H), 1.40–1.20 (m, 13 H), 0.9 (t, 3 H, *J* = 7.0 Hz), 0.83 (d, 3 H, *J* = 6.0 Hz); ^{13}C NMR (125 MHz, $CDCl_3$, δ_C) 169.8, 166.4, 166.1, 165.2, 137.1, 133.6, 133.5, 133.1, 129.9, 129.8, 129.7, 129.6, 129.1, 129.1, 128.9, 128.5, 128.4, 128.3, 128.0, 126.1, 109.4, 104.3, 101.7, 98.2, 98.1, 83.4, 81.6, 80.0, 78.6, 77.8, 76.7, 76.2, 75.0, 69.0, 68.9, 68.3, 65.9, 64.4, 63.2, 53.6, 31.8, 29.4, 29.3, 29.2, 27.9, 26.3, 26.2, 23.4, 22.6, 17.1, 14.1. HRMS (ESI) calcd. for $(M+Na)^+$ $C_{59}H_{71}NO_{18}Na$ 1104.4563, found 1104.4565.

Octyl 2,3,5-tri-*O*-benzoyl-6-*O*-levulinoyl-β-D-galactofuranosyl-(1→5)-2,3,6-tri-*O*-benzoyl-β-D-galactofuranosyl-(1→4)-2,3-isopropylidene-α-L-rhamnopyranosyl-(1→3)-2-acetamido-4,6-*O*-benzylidene-2-deoxy-α-D-glucopyranoside (13).

The compound was prepared from alcohol **12** (2.3 g, 2.1 mmol), thioglycoside **10** (1.8 g, 2.5 mmol), powdered 4 Å molecular sieves (0.5 g), NIS (0.9 g, 4.0 mmol), silver trifluoromethane sulfonate (0.13 g, 0.5 mmol) in CH_2Cl_2 (80 mL) as described for compound **11**. TMSOTf (10 μL) was added 10 min after AgOTf addition as the glycosylation was very sluggish. Tetrasaccharide **13** (3.1 g, 89%) was obtained as an amorphous solid. R_f 0.31, (1:1, hexane–EtOAc); $[\alpha]_D$ –6.7 (*c* 0.5, $CHCl_3$); ^{1}H NMR (500 MHz, $CDCl_3$, δ_H) 8.01–7.91 (m, 9 H, Ar), 7.83–7.74 (m, 4 H, Ar), 7.60–7.03 (m, 22 H), 5.83 (ddd, 1 H, *J* = 3.9, 7.7, 11.4 Hz), 5.80–5.76 (m, 3 H), 5.74 (s, 1 H), 4.64 (d, 1 H, *J* = 3.5 Hz), 5.57–5.53 (m, 3 H), 5.10 (s, 1 H), 4.88 (dd, 1 H, *J* = 3.7, 4.9 Hz), 4.74–4.66 (m, 3 H), 4.54 (dd, 1 H, *J* = 4.0, 11.9 Hz), 4.46–4.34 (m, 3 H), 4.30–4.26 (m, 2 H), 4.09 (d, 1 H, *J* = 5.9 Hz), 4.02–3.82 (m, 3 H), 3.79–3.66 (m, 2 H), 3.64–3.53 (m, 2 H), 3.41 (ddd, 1 H, *J* = 6.6, 9.6, 13.2 Hz), 2.61–2.40 (m, 4 H), 2.03 (s, 6 H), 1.64–1.58 (m, 3 H), 1.52 (s, 3 H), 1.40–1.20 (m, 13 H), 0.9 (t, 3 H, *J* = 7.0 Hz), 0.83 (d, 3 H,

J = 6.0 Hz); ^{13}C NMR (125 MHz, $CDCl_3$, δ_C) 206.2, 172.1, 169.9, 166.1, 165.7, 165.3, 165.2, 137.1 133.4, 133.3, 133.2, 133.2, 133.1, 133.0, 129.9, 129.9, 129.8, 129.8, 129.7, 129.6, 129.5, 129.0, 129.0, 128.9, 128.8, 128.7, 128.5, 128.3, 128.2, 128.0, 126.2, 109.4, 105.5, 104.0, 101.6, 98.2, 98.1, 82.7, 82.0, 81.9, 81.9, 80.1, 77.8, 77.7, 77.6, 76.4, 76.2, 74.8, 72.9, 70.4, 68.9, 68.3, 64.8, 64.4, 63.2, 63.1, 53.6, 37.8, 31.8, 29.6, 29.5, 29.3, 29.3, 29.2, 27.9, 27.8, 26.3, 26.2, 23.4, 22.6, 17.2, 14.1. HRMS (ESI) calcd. for $(M+Na)^+$ $C_{91}H_{99}NO_{28}Na$ 1676.6245, found 1676.6241.

Octyl 2,3,5-tri-*O*-benzoyl-β-D-galactofuranosyl-(1→5)-2,3,6-tri-*O*-benzoyl-β-D-galactofuranosyl-(1→4)-2,3-isopropylidene-α-L-rhamnopyranosyl-(1→3)-2-acetamido-4,6-*O*-benzylidene-2-deoxy-α-D-glucopyranoside (14).

To a solution of **13** (2.86 g, 1.7 mmol) in CH_2Cl_2–MeOH (9:1, 90 mL), hydrazine acetate (0.4 g, 4.3 mmol) was added and the mixture was stirred for 100 min. The solution was poured into chilled H_2O (30 mL) and the CH_2Cl_2 layer was separated, washed with H_2O (30 mL), separated again, dried with anhydrous Na_2SO_4 and concentrated to a syrup that was purified by column chromatography (2:1 hexane–EtOAc) to afford **14** (2.6 g, 97%) as an amorphous solid. R_f 0.40 (1:1 hexane–EtOAc); HRMS (ESI) calcd. for $(M+Na)^+$ $C_{86}H_{93}NO_{26}Na$ 1578.5878, found 1578.5856. The compound was not further characterized before being carried onto the next step.

Octyl 2,3,6-tri-*O*-benzoyl-5-*O*-levulinoyl-β-D-galactofuranosyl-(1→6)-2,3,5-tri-*O*-benzoyl-β-D-galactofuranosyl-(1→5)-2,3,6-tri-*O*-benzoyl-β-D-galactofuranosyl-

(1→4)-2,3-isopropylidene-α-L-rhamnopyranosyl-(1→3)-2-acetamido-4,6-*O*-benzylidene-2-deoxy-a-D-glucopyranoside (15).

The compound was prepared from alcohol **14** (2.6 g, 1.67 mmol), thioglycoside **9** (1.4 g, 2.0 mmol), powdered 4 Å molecular sieves (0.65 g), NIS (0.54 g, 2.4 mmol), silver trifluoromethane sulfonate (0.13 g, 0.5 mmol) in CH_2Cl_2 (80 mL) as described for compound **11**. TMSOTf (10 μL) was added 10 min after AgOTf addition as the glycosylation was very sluggish. Pentasaccharide **15** (2.86 g, 81%) was obtained as an amorphous solid. R_f 0.15 (1:1, hexane–EtOAc), $[\alpha]_D$ +2.1 (*c* 0.4, $CHCl_3$); ^{1}H NMR (500 MHz, $CDCl_3$, δ_H) 8.01–7.91 (m, 15 H), 7.88–7.76 (m, 5 H), 7.60–7.04 (m, 30 H), 5.90 (ddd, 1 H, *J* = 3.8, 7.7, 11.6 Hz), 5.82 (d, 1 H, *J* = 5.3 Hz), 5.78 (s, 1 H), 5.76–5.70 (m, 3 H), 5.66–5.62 (m, 2 H), 5.55 (d, 1 H, *J* = 1.5 Hz), 5.54 (s, 1 H), 5.43 (d, 1 H, *J* = 4.9 Hz), 5.33 (d, 1 H, *J* = 1.5 Hz), 5.19 (s, 1 H), 5.10 (s, 1 H), 4.84 (dd, 1 H, *J* = 4.2, 4.2 Hz), 4.74–4.64 (m, 4 H), 4.63-4.50 (m, 2 H), 4.55–4.50 (m, 1 H), 4.44–4.38 (m, 2 H), 4.31–4.26 (m, 2 H), 4.12–4.07 (m, 2 H), 4.02–3.82 (m, 3 H), 3.78–3.67 (m, 2 H), 3.62 (dd, 1 H, *J* = 9.6, 9.6 Hz), 3.55 (dd, 1 H, *J* =7.2, 10.2 Hz), 3.41 (ddd, 1 H, *J* = 6.6, 9.6, 13.2 Hz), 2.63–2.40 (m, 4 H), 2.02 (s, 3 H), 2.01 (s, 3 H), 1.64–1.58 (m, 3 H), 1.53 (s, 3 H), 1.40–1.20 (m, 13 H), 0.9 (t, 3 H, *J* = 7.0 Hz), 0.83 (d, 3 H, *J* = 6.0 Hz); ^{13}C NMR (125 MHz, $CDCl_3$, δ_C) 205.9, 171.9, 169.8, 166.1, 165.9, 165.8, 165.7, 165.5, 165.4, 165.3, 165.2, 164.9, 137.1, 133.4, 133.3, 133.3, 133.2, 133.1, 133.1, 132.9, 132.8, 130.0, 129.9, 129.8, 129.7, 129.6, 129.6, 129.6, 129.1, 129.0, 129.0, 128.9, 128.9, 128.8, 128.5, 128.4, 128.3, 128.2, 128.2, 128.2, 128.0, 126.2, 109.4, 106.7, 105.8, 104.0, 101.7, 98.2, 98.1, 82.7, 82.6, 82.0, 81.8, 81.6, 81.5, 80.1, 77.8, 77.8, 77.6, 76.3, 76.2, 74.9, 73.0, 71.8, 70.2, 68.9, 68.3, 67.7, 65.0, 64.4, 63.5, 63.2, 53.6, 37.9, 31.8, 29.6, 29.5, 29.4, 29.3, 29.2, 28.0, 27.9, 26.4, 26.2, 23.4, 22.6, 17.2, 14.1. HRMS (ESI) calcd. for $(M+2Na)^{2+}$ $C_{118}H_{121}NO_{36}Na_2$ 1087.3743, found 1087.3740.

Supplementary References

1. **Lee RE, Brennan PJ, Besra GS.** 1997. Mycobacterial arabinan biosynthesis: the use of synthetic arabinoside acceptors in the development of an arabinosyl transfer assay. Glycobiology **7:**1121-1128.
2. **Auzanneau FI, Hanna HR, Bundle DR.** 1993. The synthesis of chemically modified disaccharide derivatives of the Shigella flexneri Y polysaccharide antigen. Carbohydr Res **240:**161-181.
3. **Auzanneau FI, Bundle DR.** 1993. Synthesis of chlorodeoxy trisaccharides related to the Shigella flexneri Y polysaccharide. Carbohydr Res **247:**195-209.
4. **Konradsson P, Udodong UE, Fraser-Reid B.** 1990. Iodonium promoted reactions of disarmed thioglycosides. Tetrahedron Letters **31:**4313-4316.
5. **Pozsgay V, Jennings HJ.** 1988. Synthetic oligosaccharides related to group B streptococcal polysaccharides. 3. Synthesis of oligosaccharides corresponding to the common polysaccharide antigen of group B streptococci. The Journal of Organic Chemistry **53:**4042-4052.
6. **Aguilera B, Romero-Ramirez L, Abad-Rodriguez J, Corrales G, Nieto-Sampedro M, Fernandez-Mayoralas A.** 1998. Novel disaccharide inhibitors of human glioma cell division. J Med Chem **41:**4599-4606.
7. **Hamer GK, Perlin AS.** 1976. A 13C-n.m.r. spectral study of chondroitin sulfates A, B, and C: evidence of heterogeneity. Carbohydrate Research **49:**37-48.
8. **Completo GC, Lowary TL.** 2008. Synthesis of Galactofuranose-Containing Acceptor Substrates for Mycobacterial Galactofuranosyltransferases. The Journal of Organic Chemistry **73:**4513-4525.
9. **Cyr N, Perlin AS.** 1979. The conformations of furanosides. A 13C nuclear magnetic resonance study. Canadian Journal of Chemistry **57:**2504-2511.
