# Supplementary figures and images for: Lcp1 Is a Phosphotransferase Responsible for Ligating Arabinogalactan to Peptidoglycan in Mycobacterium tuberculosis
Source: mBio. 2016 Aug 2;7(4):e00972-16. doi: 10.1128/mBio.00972-16 (PMC4981717; doi:10.1128/mBio.00972-16)

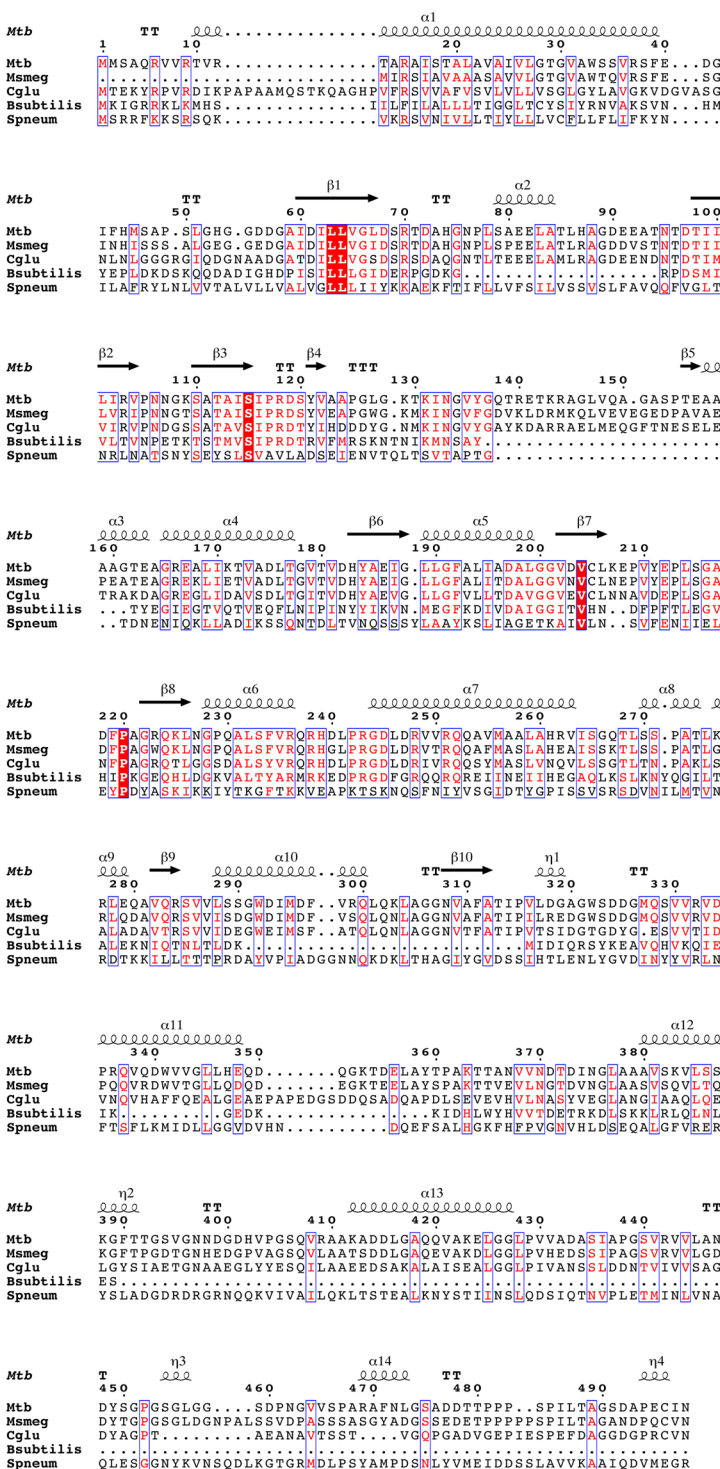

Supplement: Figure S1 — Sequence alignment of Lcp1 orthologues from M. tuberculosis, M. smegmatis, and C. glutamicum and LCP homologs from B. subtilis and S. pneumoniae. Amino acid sequences were aligned using ClustalW and rendered with EsPRIPT. Secondary structure information was obtained from PDB coordinates 2XXP. Download [file mbo004162922sf1.pdf]

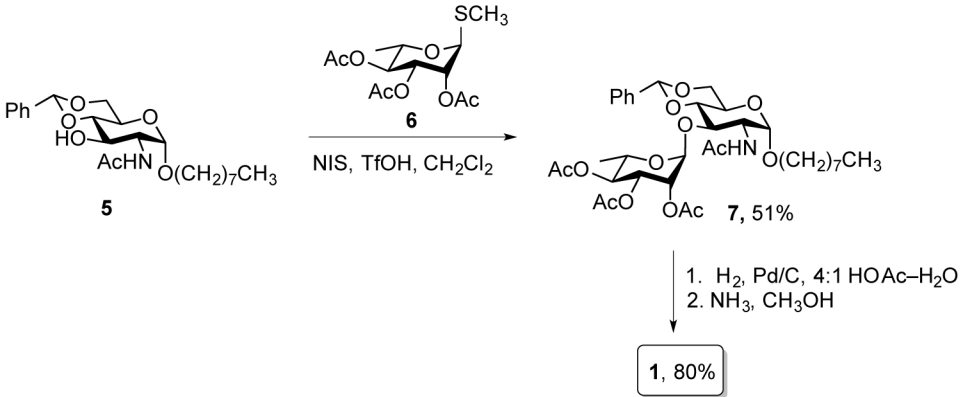

Supplement: Figure S2 — Reaction scheme for the synthesis of octyl α-l-rhamnopyranosyl-(1→3)-2-acetamido-2-deoxy-α-d-glucopyranoside (compound 1). Download [file mbo004162922sf2.pdf]

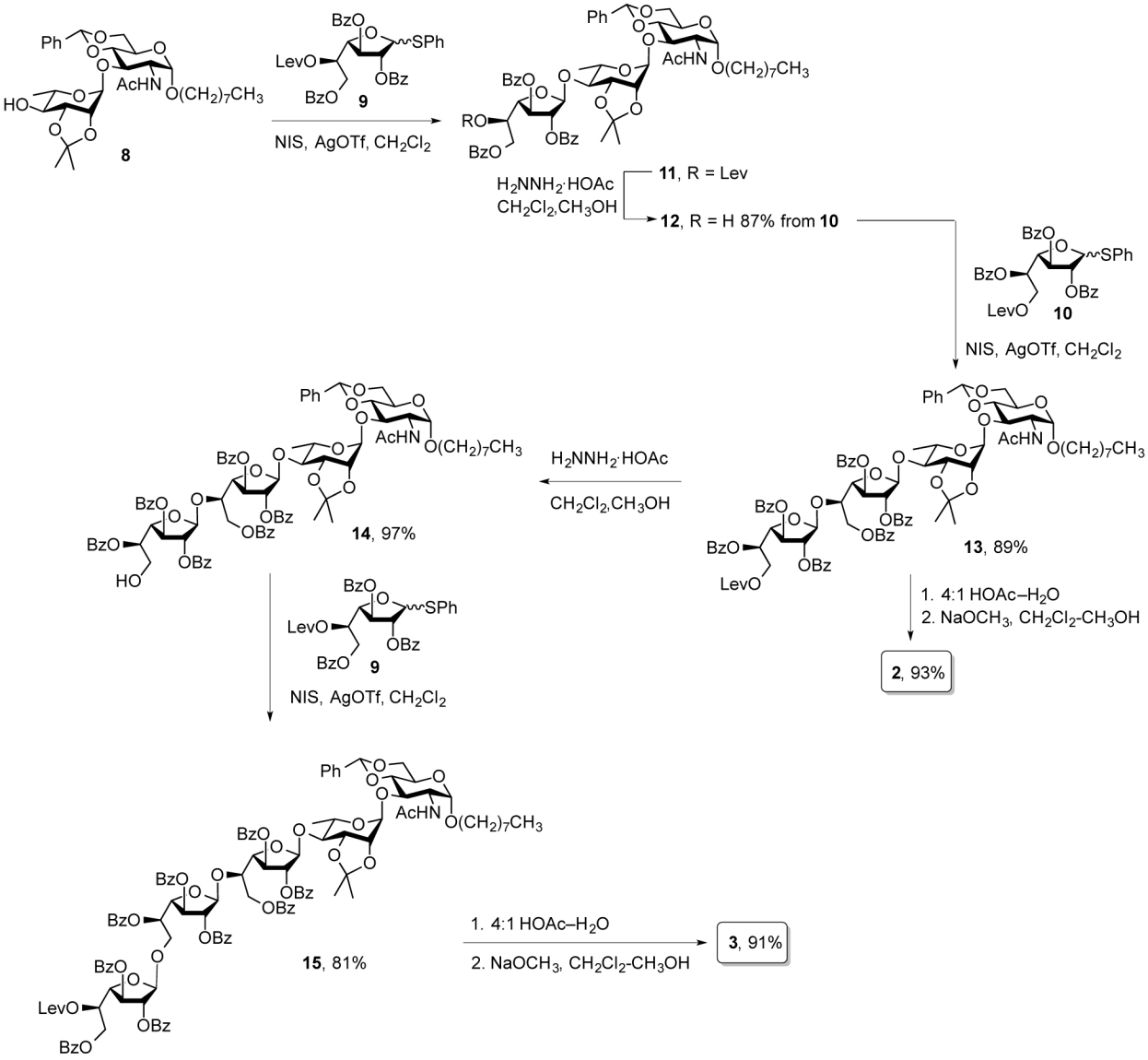

Supplement: Figure S3 — Reaction scheme for the synthesis of octyl-β-d-galactofuranosyl-(1→5)-β-d-galactofuranosyl-(1→4)-α-l-rhamnopyranosyl-(1→3)-2-acetamido-2-deoxy-α-d-glucopyranoside (compound 2) and octyl-β-d-galactofuranosyl-(1→6)-β-d-galactofuranosyl-(1→5)-β-d-galactofuranosyl-(1→4)-α-l-rhamnopyranosyl-(1→3)-2-acetamido-2-deoxy-α-d-glucopyranoside (compound 3). Download [file mbo004162922sf3.pdf]

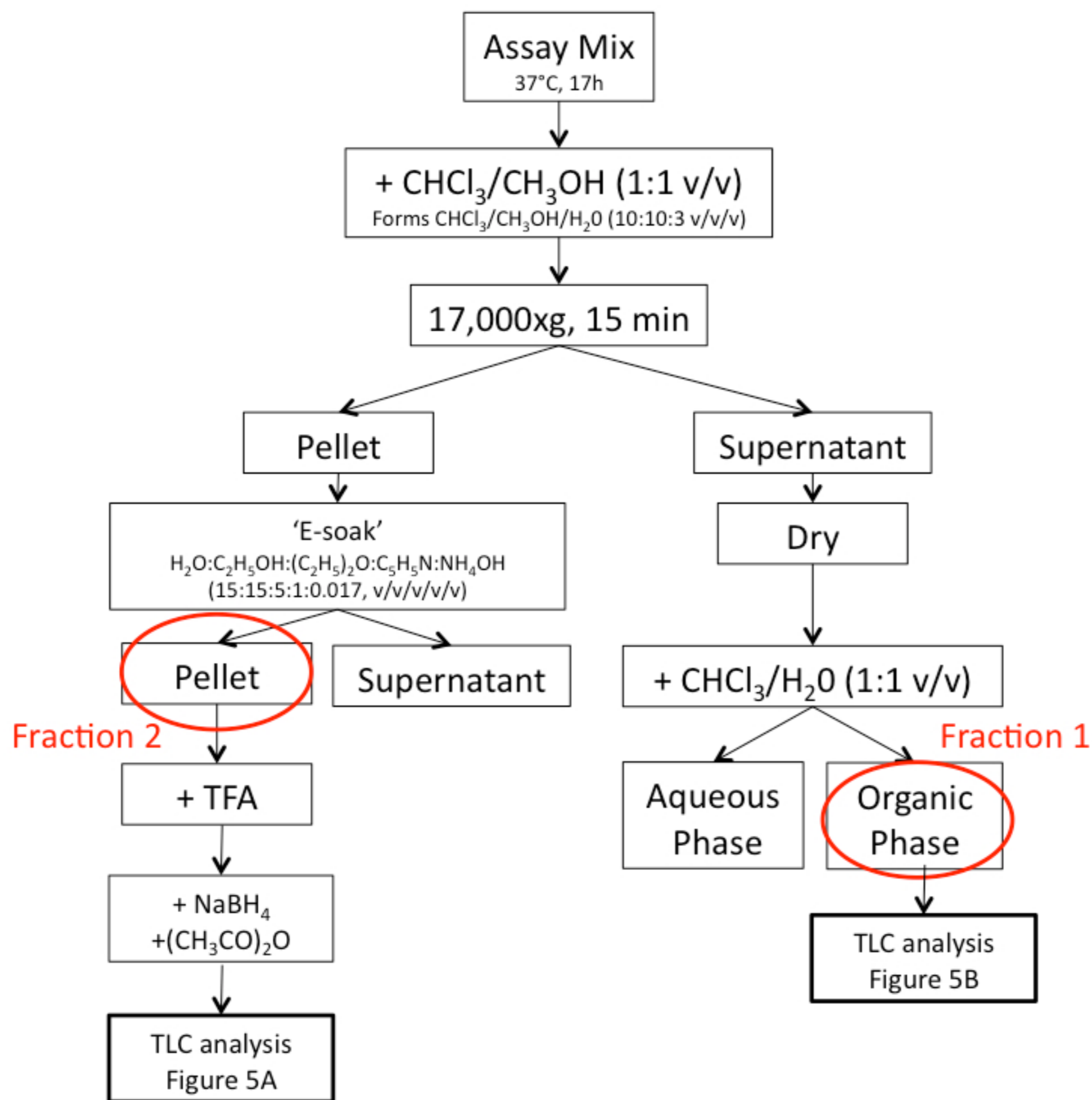

Supplement: Figure S4 — Flow chart tracking radioactivity incorporated from UDP-[14C]Galp through each of the analytical steps leading to TLC analysis (Fig. 5). Download [file mbo004162922sf4.pdf]
